# Persistent socio-demographic inequalities in depression among older adults in China during and after COVID-19: a systematic review and meta-analysis

**DOI:** 10.3389/fpubh.2026.1929179

**Published:** 2026-09-16

**Authors:** Nili Wang, Xin Lan, Jun Zhong, Xiaojing Fan, Yuan Xing, Shaonong Dang, Zhenjie Wang

**Affiliations:** 1 School of Sociology, Beijing Normal University, Beijing, China; 2 School of Public Health, Xi'an Medical University, Xi’an, Shaanxi, China; 3 School of Public Health, Peking University Health Science Center, Beijing, China; 4 School of Public Policy and Administration, Xi'an Jiaotong University, Xi’an, Shaanxi, China; 5 School of Public Health, Xi'an Jiaotong University Health Science Center, Xi’an, Shaanxi, China; 6 Department of Science Research, China Population Association, Beijing, China

**Keywords:** China, COVID-19 pandemic, depressive symptoms, health inequalities, older adults

## Abstract

**Objective:**

To examine socio-demographic inequalities in depressive symptoms among older adults in China during and after the COVID-19 pandemic and assess whether these inequalities remained stable or changed over time.

**Methodology:**

This systematic review and meta-analysis followed PRISMA guidelines. Nine English and Chinese databases were searched from 1 January 2020 to 15 June 2026. Eligible studies were conducted among community-dwelling adults aged ≥60 years in mainland China, used probability sampling and standardized measures of depressive symptoms, and reported cross-sectional data. Study quality was assessed using the 11-item Agency for Healthcare Research and Quality checklist. Potential publication bias was assessed using Begg’s and Egger’s tests and trim-and-fill analysis. Year-stratified prevalence and pooled odds ratios (ORs) were estimated for disparities by gender, marital status, urban–rural residence, living arrangement and educational attainment.

**Results:**

Seventy-nine studies involving 199,116 older adults from 21 provincial-level regions were included; 52 studies were rated as high quality and 27 as moderate quality, with none rated as low quality. Pooled prevalence fluctuated across investigation years, from 16.6% (95% CI 12.1–22.4) in 2022 to 22.9% (95% CI 18.3–28.3) in 2024–2025, with substantial between-study heterogeneity (overall I^2^ = 99.55%). Higher odds of depressive symptoms were observed among women, unmarried older adults, rural residents, those living alone and those with lower educational attainment. The largest disparities were related to education (OR 1.693, 95% CI 1.388–2.060), living arrangement (OR 1.671, 95% CI 1.437–1.945) and marital status (OR 1.652, 95% CI 1.467–1.860). Year-level analyses suggested limited temporal variation overall: marital-status-related inequality showed a small upward trend, whereas living-arrangement-related inequality showed a non-significant tendency toward attenuation. No broad systematic restructuring was observed.

**Conclusion:**

Depressive symptoms among older adults in China fluctuated across the pandemic and post-pandemic periods, while socio-demographic inequalities remained broadly stable. Major public health disruption appeared more likely to expose and prolong pre-existing inequalities than fundamentally reshape them in the short term. These findings provide a useful comparative reference for understanding mental health vulnerability in aging societies facing public health emergencies.

**Systematic review registration:**

https://www.crd.york.ac.uk/PROSPERO/view/CRD42025642200; identifier: CRD42025642200.

## 1 Introduction

Depression is a major contributor to the global burden of mental disorders and an important public health challenge in aging societies worldwide (1, 2). Reported prevalence of depression among older adults varies substantially across populations and studies. Recent global meta-analyses have reported pooled prevalence estimates of 31.74 and 35.1% (2, 3), with substantial variation across individual studies and population settings. In China, reported estimates have similarly varied widely, ranging from 3.8 to 50.32% (4, 5). Existing evidence consistently suggests that older adults who are female, live in rural areas, are unmarried, live alone, or have lower educational attainment are at higher risk of depression (6–10). These disparities are commonly linked to unequal access to social resources, social support and cumulative life-course disadvantage (11–13). Nevertheless, such patterns are not fully consistent across contexts. Cross-national evidence has shown substantial variation in socio-demographic inequalities in depression (6), and some studies have reported attenuation or even reversal of specific risk structures in particular regions or periods (14, 15). For example, a cross-sectional study of 1,173 community-dwelling older adults in Hunan, China, found no significant association between gender and depressive symptoms (16). Together, these findings suggest that the social patterning of late-life depression may be context-dependent rather than entirely static.

Major public health emergencies provide a distinctive context in which existing health inequalities may be exposed or amplified (17). Since the COVID-19 outbreak, a growing body of research has examined depression among older adults, but reported prevalence estimates remain highly heterogeneous, ranging from 2.79 to 37.34% (17–20). China, as the first country to experience the COVID-19 outbreak (18, 19), provides an informative setting for examining whether socio-demographic inequalities in depression among older adults persist or change under major public health disruption. Its very large and rapidly aging population is characterized by substantial geographic and socioeconomic disparities in access to health-related resources and public health services (21, 22). At the same time, China’s COVID-19 response underwent successive policy adjustments across the pandemic and post-pandemic periods, creating a changing social and public health environment (23). However, existing studies in China have focused mainly on overall prevalence estimates or pandemic-related temporal changes in depressive symptoms (18–20, 24, 25). Systematic evidence on whether socio-demographic inequalities in depression persisted or changed across these periods remains limited (26).

This study aimed to examine socio-demographic inequalities in depressive symptoms among older adults in China during and after the COVID-19 pandemic. Specifically, we assessed disparities by gender, marital status, residence, living arrangement and educational attainment, and explored whether these inequalities remained stable or changed across investigation years. By synthesizing evidence across regions and periods, this study aimed to inform the identification of high-risk groups in China and to provide comparative evidence for research on late-life depression inequalities in aging societies.

## 2 Methods

### 2.1 Study design and registration

This systematic review was registered with PROSPERO (CRD42025642200) and conducted in accordance with the Preferred Reporting Items for Systematic Reviews and Meta-Analyses (PRISMA) 2020 statement (27). The research question and eligibility criteria were structured using the PICOS framework, as detailed below, and the PROSPERO record was updated to reflect the final review scope, eligibility criteria, search period and analysis plan.

### 2.2 Search strategy and data sources

To capture the most up-to-date evidence, we adopted a rolling search strategy and updated the database search approximately every 2 weeks until the final search on 15 June 2026. The search covered studies conducted from 1 January 2020, when WHO initiated its organizational emergency response to COVID-19 (19), to the same cutoff date. We searched PubMed, Embase, Web of Science, Scopus, PsycINFO, CNKI, Wanfang Data, CQVIP and CBMdisc. Search terms combined concepts related to older adults, depressive symptoms and China. The full search strategy for each database is provided in Supplementary Table 2. We also screened the reference lists of eligible studies as a supplementary search strategy; no additional eligible studies were identified.

### 2.3 Eligibility criteria

Eligibility criteria were developed using the PICOS framework, which encompasses Population, Intervention, Comparator, Outcome, and Study Design (26). We included studies conducted among community-dwelling older adults aged 60 years or older in mainland China, based on general-population samples and probability sampling methods, that assessed depressive symptoms using standardized measurement instruments and reported prevalence data collected after 1 January 2020. Studies focusing on specialized populations, including institutionalized older adults, inpatients, or populations defined by specific diseases or disaster exposures, were excluded. We also excluded studies without clear measurement tools or prevalence data, non-probability sampling studies, reviews, conference papers, case reports, editorials, comments, letters and unpublished manuscripts. Where multiple reports were based on the same dataset, only the most complete and recent study was retained.

### 2.4 Study selection

All retrieved records were imported into Microsoft Excel and screened in four stages by two reviewers (ZW and NW). After deduplication, titles and abstracts were screened for relevance to the target population and study design, followed by full-text review against the eligibility criteria. Disagreements were resolved through discussion, with consultation from additional reviewers (JZ, XF, YX and SD) when necessary. The study selection process is shown in the PRISMA flow diagram (Figure 1).

**Figure 1:**
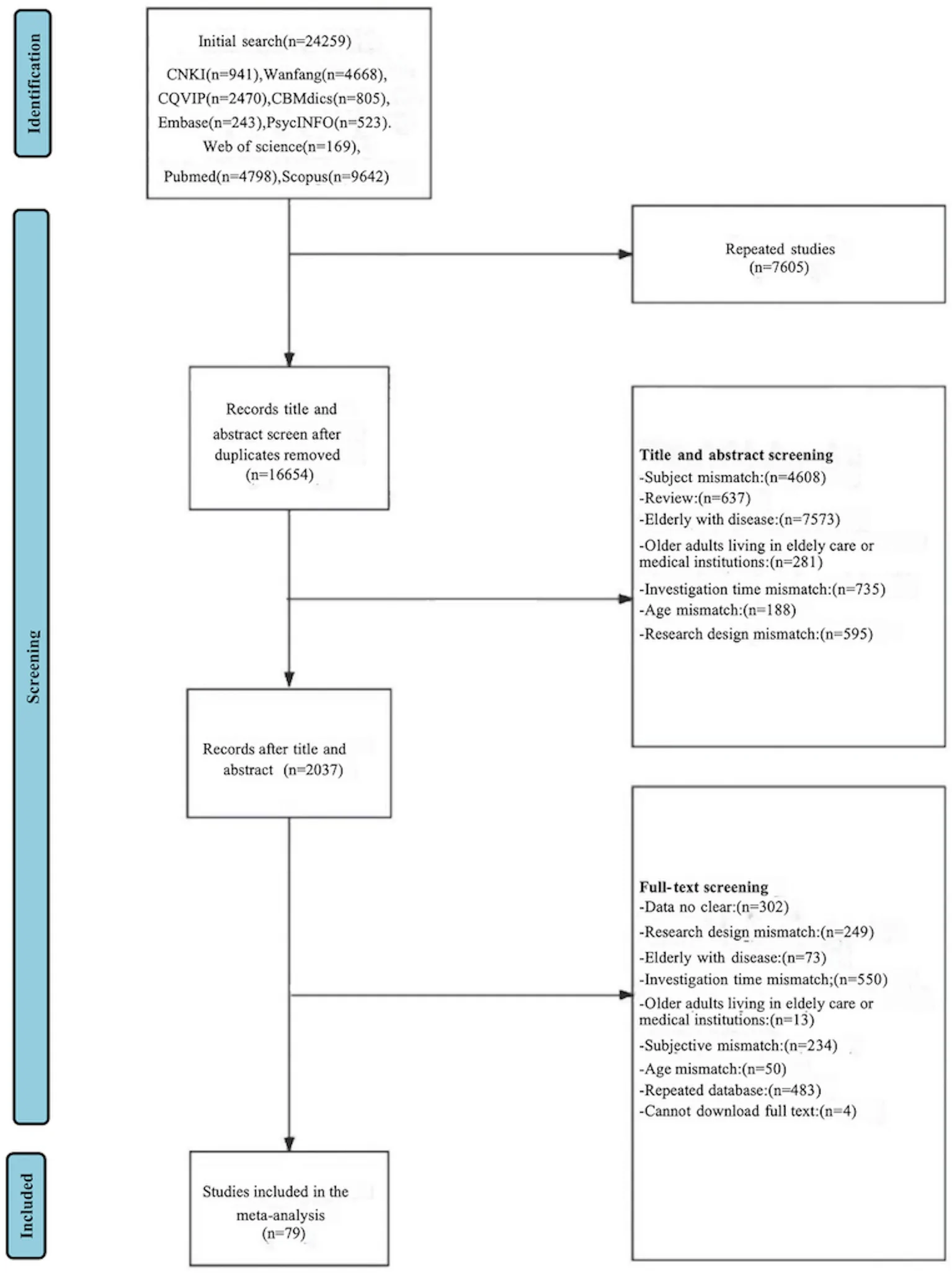
Flow diagram of study selection for the meta-analysis.

### 2.5 Data extraction

Two reviewers (NW and XL) independently extracted data using a standardized data extraction form. Extracted information included study characteristics (author, publication year, survey year, region, sampling method, sample size and measurement instrument), overall prevalence of depressive symptoms, and subgroup data by gender, urban–rural residence, marital status, living arrangement and educational attainment where available. All extracted data were cross-checked twice to ensure accuracy.

### 2.6 Quality assessment

Study quality was assessed using the 11-item checklist recommended by the Agency for Healthcare Research and Quality (AHRQ) (28). Given that the evidence synthesized in this review was derived primarily from cross-sectional prevalence studies, the AHRQ checklist was selected because of its applicability to the methodological appraisal of cross-sectional studies (29). Each item was assigned one point if the criterion was met, yielding a total score ranging from 0 to 11; scores of 0–3, 4–7 and 8–11 were classified as low, moderate and high quality, respectively.

### 2.7 Statistical analysis

All quantitative syntheses were performed using Stata (version 18.0; StataCorp, College Station, TX, USA). Study-specific proportions of depressive symptoms were synthesized using random-effects models to account for substantial between-study heterogeneity and the expectation that true prevalence may vary across populations, settings and measurement approaches (30). Sensitivity analyses using the Freeman–Tukey double arcsine transformation for prevalence pooling were conducted to assess the robustness of year-stratified prevalence estimates. Heterogeneity was assessed using Cochran’s Q test and the I^2^ statistic (31). High I^2^ values are common in prevalence meta-analyses and were therefore interpreted alongside subgroup and meta-regression analyses rather than as a reason to preclude quantitative synthesis (32). Pooled prevalence estimates are presented with 95% confidence intervals (CIs) and were stratified by investigation year. For year-stratified analyses, adjacent later years were combined when the number of eligible studies within a single year was too small to support stable pooled estimates. Accordingly, 2024 and 2025 were combined in the prevalence analysis, and 2023–2024 estimates were combined for some year-stratified OR analyses. Univariable random-effects meta-regression was further conducted as an exploratory analysis to examine study-level sources of prevalence variation.

To describe subgroup patterns, subgroup analyses of pooled prevalence were conducted by investigation year, geographic region, gender, marital status, urban–rural residence, living arrangement, and educational attainment (junior secondary school or below vs. senior secondary school and above). Detailed results are provided in the Supplementary material.

To quantify socio-demographic inequalities in depressive symptoms, study-level 2 × 2 tables were constructed from the reported subgroup counts, and crude odds ratios (ORs) were pooled using random-effects models. Where applicable, ORs were further stratified by investigation year to examine temporal variations in inequality across the pandemic and post-pandemic phases. Differences between year strata were formally assessed using between-stratum heterogeneity statistics.

To explore temporal shifts in inequality, we conducted a secondary year-level trend analysis based on year-specific pooled ORs for each socio-demographic comparison. Because ratio effect measures are conventionally analyzed on the logarithmic scale, pooled ORs were transformed to ln(OR) prior to regression analysis (33). Similar applications of weighted regression to log-transformed odds ratios have been reported in previous meta-analytic research (34). Investigation year was then entered as a continuous predictor in an inverse-variance weighted linear regression, with weights derived from the variance of the year-specific pooled ln(OR). Standard errors for year-specific pooled ln(OR) were reconstructed from the corresponding 95% CIs on the log scale using standard methods (35, 36). Regression coefficients were exponentiated and expressed as exp.(β), representing the ratio of odds ratios (ROR) per year, so that the estimated slope could be interpreted on the original OR scale as the multiplicative change in the year-specific pooled OR associated with a 1-year increase in investigation year (37). Because this analysis was based on year-specific pooled estimates rather than study-level or individual-level observations, it was treated as an exploratory ecological analysis.

Potential small-study effects were assessed on the logit-transformed prevalence scale by visual inspection of funnel plot asymmetry, Begg’s rank correlation test and Egger’s regression test. Trim-and-fill analysis was further conducted to evaluate the potential influence of funnel plot asymmetry on the pooled prevalence estimate. Leave-one-out sensitivity analyses were subsequently conducted to evaluate the influence of individual studies on the pooled prevalence estimate and between-study heterogeneity.

## 3 Results

### 3.1 Literature search

A total of 24,259 records were identified across nine databases. After removal of 7,605 duplicates, 16,654 records were screened by title and abstract, and 2,037 full-text articles were assessed for eligibility. Ultimately, 79 studies met the inclusion criteria and were included in the final analysis (Figure 1).

### 3.2 Study characteristics and quality assessment

This review included 79 eligible studies conducted across 21 provincial-level regions in mainland China, including two mixed multi-province samples, encompassing a total of 199,116 participants aged 60 years and above. Survey years ranged from 2020 to 2025 (5, 16, 38–114), and individual study sample sizes ranged from 165 to 23,956 participants. All studies used probability sampling and standardized instruments to assess depressive symptoms. The most commonly used instruments were PHQ measures (*n* = 29), GDS measures (*n* = 28), and the CES-D (*n* = 14), while the remaining studies used other standardized instruments. Subgroup-specific data were available from 34 studies for gender, 21 for urban–rural residence, 26 for marital status, 18 for living arrangement and 19 for educational attainment. Study quality was assessed using the AHRQ tool; 27 studies were rated as moderate quality and 52 as high quality, with no studies rated as low quality (Supplementary Tables 3, 4). Additional subgroup-specific prevalence estimates are presented in Supplementary Table 5.

### 3.3 Year-stratified pooled prevalence of depressive symptoms among older adults

Between 2020 and 2025, the pooled prevalence of depressive symptoms among older adults in mainland China varied across investigation years. The pooled prevalence was 17.8% in 2020 (95% CI 12.9 to 24.1%, I^2^ = 99.56%), 20.4% in 2021 (95% CI 13.6 to 29.3%, I^2^ = 99.50%), 16.6% in 2022 (95% CI 12.1 to 22.4%, I^2^ = 99.62%), and 21.9% in 2023 (95% CI 15.8 to 29.6%, I^2^ = 99.60%). In studies conducted from 2024 to 2025, the pooled prevalence was 22.9% (95% CI 18.3 to 28.3%, I^2^ = 97.20%) (Figures 2, 3; Supplementary Table 5). Given the substantial heterogeneity, univariable random-effects meta-regression was conducted to explore study-level sources of prevalence variation. Investigation year was not significantly associated with prevalence estimates, whereas region and measurement tool showed stronger associations, particularly the higher estimates observed in studies using CES-D compared with PHQ (Supplementary Table 6).

**Figure 2:**
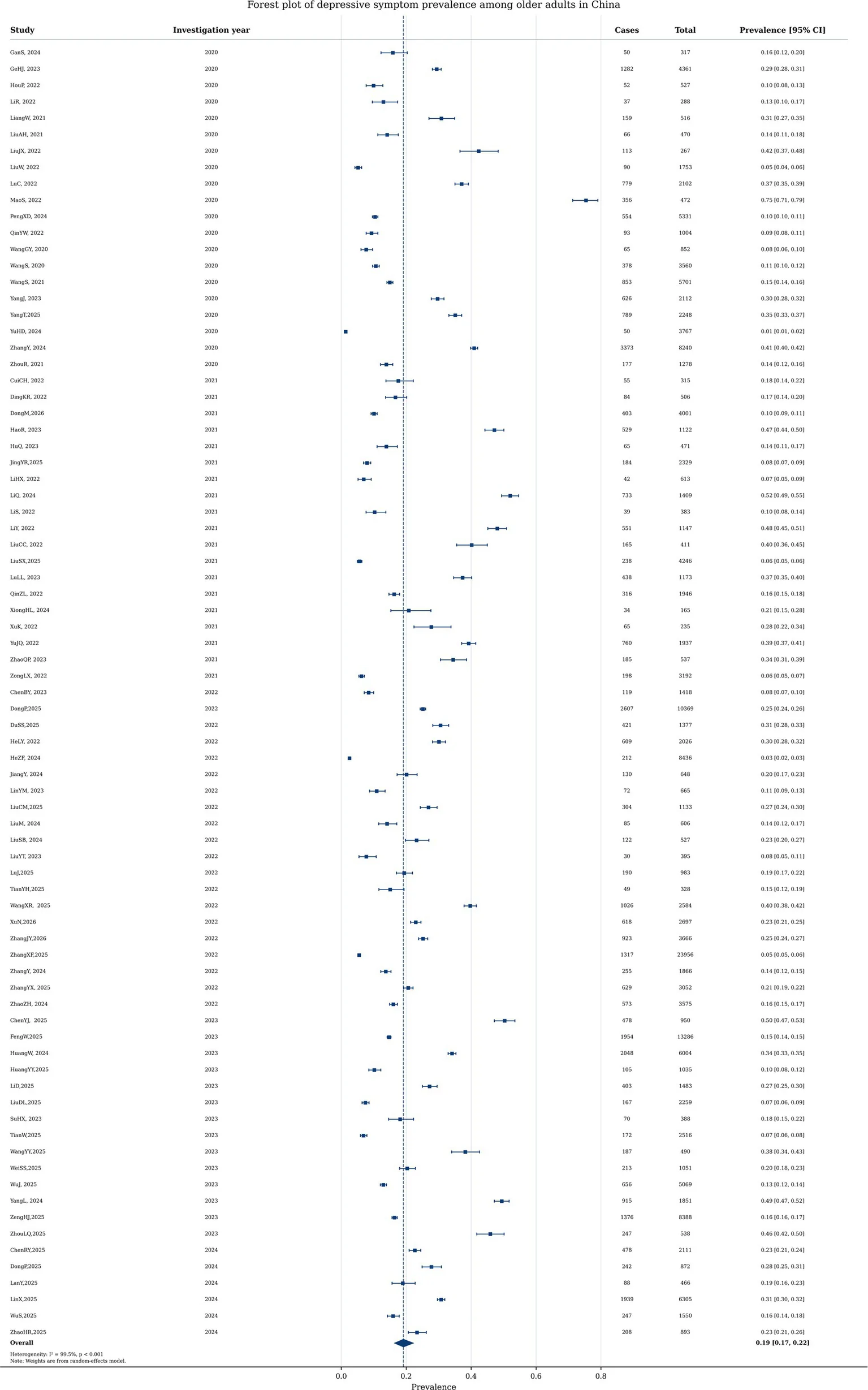
Forest plot of depressive symptom prevalence among older adults in China.

**Figure 3:**
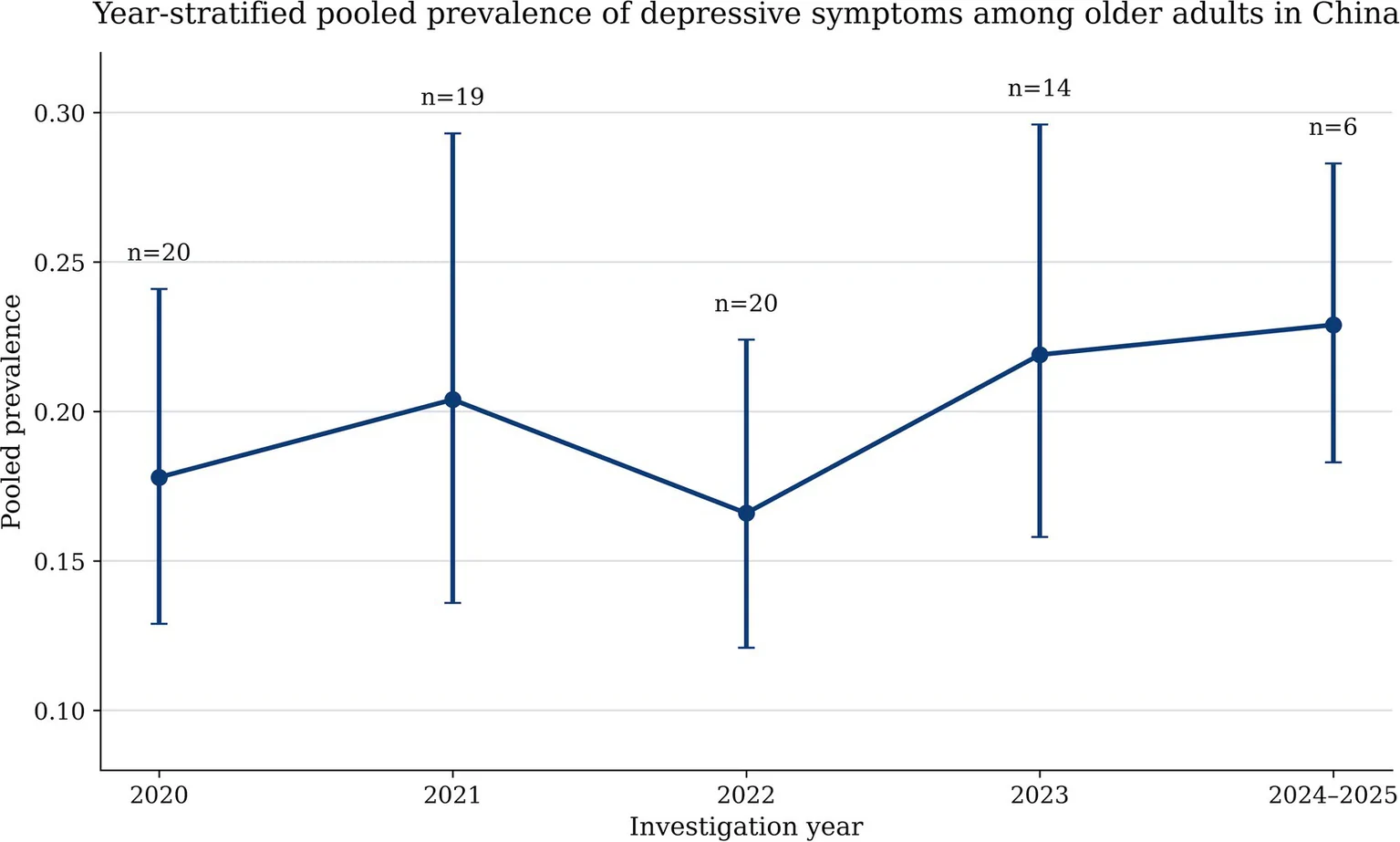
Year-stratified pooled prevalence of depressive symptoms among older adults in China. Points represent pooled prevalence estimates for each year stratum, vertical lines indicate 95% confidence intervals, and labels above the points indicate the number of included studies. The final category combined studies conducted in 2024 and 2025 because of the limited number of studies in each year.

### 3.4 Publication bias and sensitivity analysis

Visual inspection of the funnel plot suggested slight asymmetry. However, Begg’s rank correlation test (*p* = 0.059) and Egger’s regression test (*p* = 0.113) did not provide statistical evidence of small-study effects. Trim-and-fill analysis imputed no additional studies and produced the same pooled estimate as the observed analysis. Leave-one-out sensitivity analyses showed that omission of any single study did not materially alter the pooled prevalence estimate or the level of heterogeneity (Supplementary Table 9). Sensitivity analyses using the Freeman–Tukey double arcsine transformation produced slightly higher but broadly consistent year-stratified prevalence estimates, with no material change in the overall temporal pattern (Supplementary Table 10). These findings supported the overall robustness of the main results.

### 3.5 Socio-demographic disparities in depressive symptoms

Across all five socio-demographic dimensions, pooled prevalence estimates were consistently higher among disadvantaged groups than among their reference groups. Random-effects models further showed significant disparities in depression risk across all comparisons (Figure 4; Supplementary Table 7), with the largest pooled ORs observed for educational attainment (OR 1.693, 95% CI 1.388 to 2.060), living arrangement (OR 1.671, 95% CI 1.437 to 1.945) and marital status (OR 1.652, 95% CI 1.467 to 1.860), followed by gender (OR 1.476, 95% CI 1.319 to 1.662) and residence (OR 1.419, 95% CI 1.185 to 1.699), all *p* < 0.001. Detailed subgroup-specific prevalence and year-stratified OR estimates are provided in Supplementary Tables 5, 7 and Supplementary Figures 1–5.

**Figure 4:**
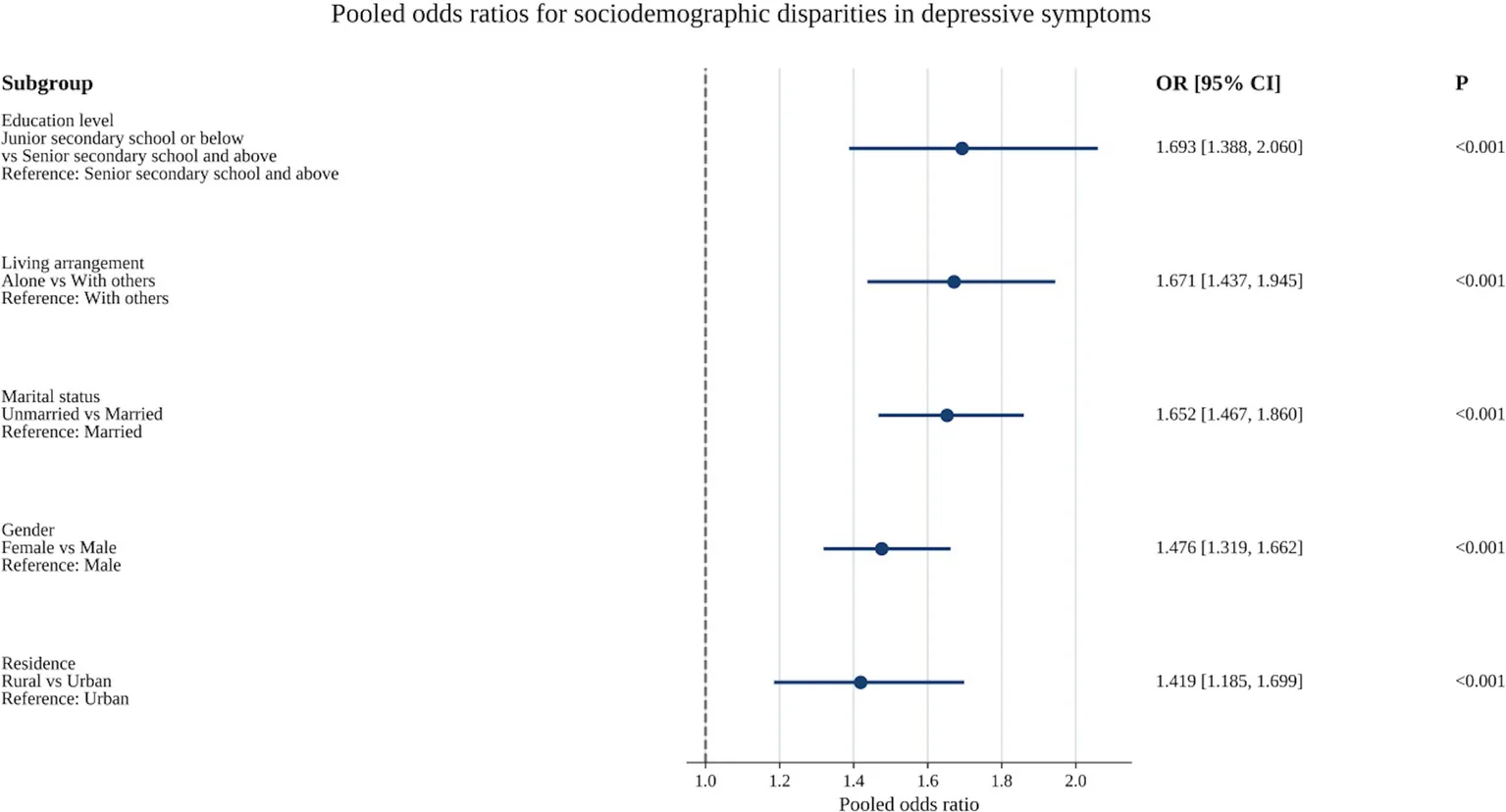
Pooled odds ratios for socio-demographic disparities in depressive symptoms among older adults in China. Points represent pooled odds ratios and horizontal lines indicate 95% confidence intervals. The vertical dashed line indicates *OR* = 1.

### 3.6 Year-level trend regression of year-specific pooled ORs

Year-level inverse-variance weighted regression of year-specific pooled ORs suggested that temporal changes in socio-demographic inequalities were generally small (Figures 5 and Supplementary Table 8). Marital-status-related inequality was the only comparison that reached conventional statistical significance, with the year-specific pooled OR increasing by an estimated 2.1% per year [exp(β) = 1.021, 95% CI: 1.016 to 1.026, *p* = 0.001]. Living-arrangement-related inequality showed a marginally non-significant tendency toward attenuation, with the year-specific pooled OR decreasing by an estimated 10.8% per year [exp(β) = 0.892, 95% CI: 0.788 to 1.009, *p* = 0.060]. No statistically significant temporal changes were observed for gender, residence or educational attainment.

**Figure 5:**
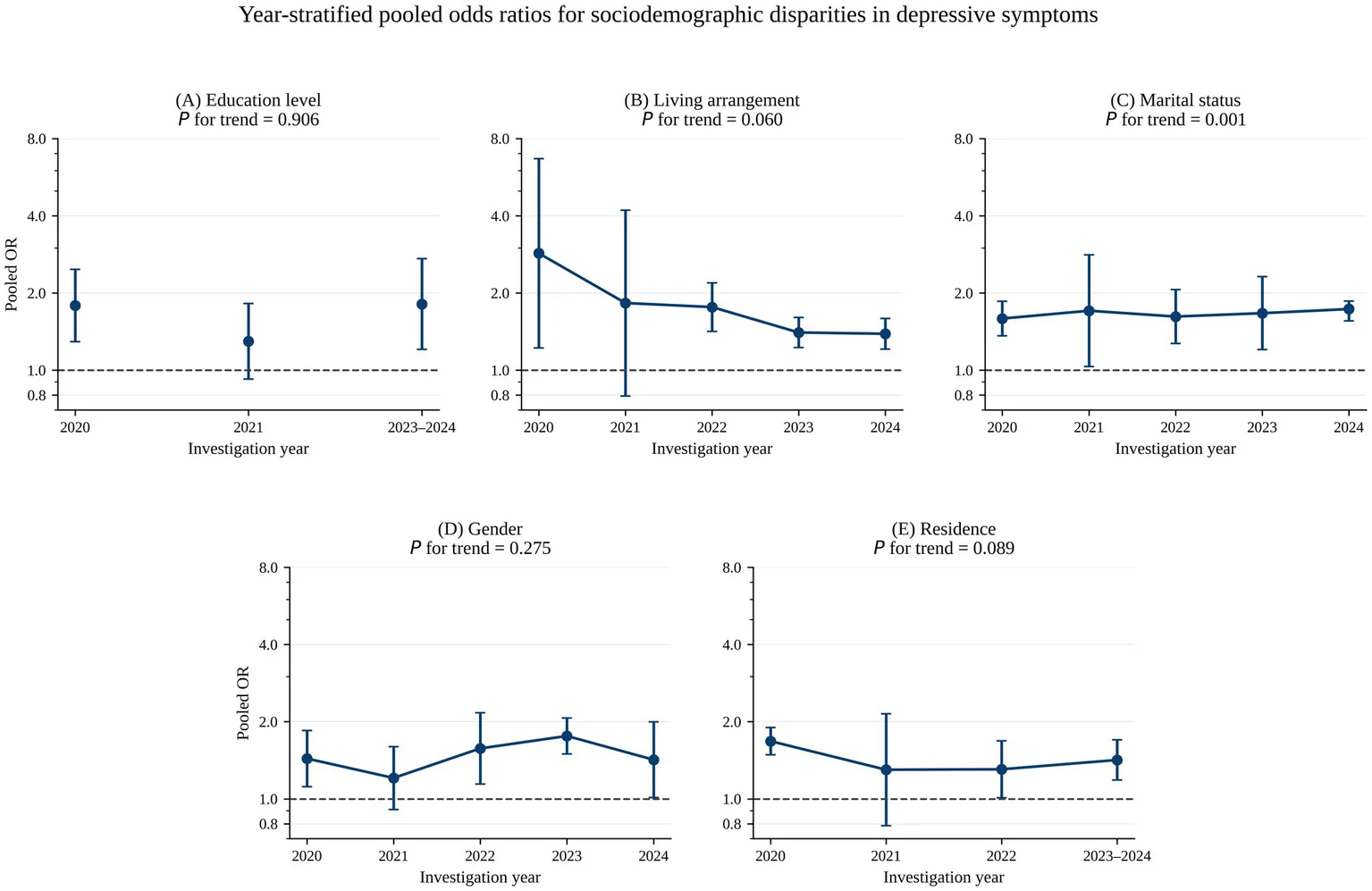
Year-stratified pooled odds ratios for socio-demographic disparities in depressive symptoms among older adults in China. Panels show disparities by **(A)** educational attainment, **(B)** living arrangement, **(C)** marital status, **(D)** gender, and **(E)** urban–rural residence. Dots indicate year-specific pooled odds ratios and vertical lines indicate 95% confidence intervals. The *y*-axis is displayed on a logarithmic scale to facilitate comparison across panels. For educational attainment, estimates for 2023 and 2024 were combined because year-specific data were sparse, and points are shown without a connecting line to avoid implying continuity across years with missing estimates. For urban–rural residence, estimates for 2023 and 2024 were also combined because year-specific data were sparse. Comparisons were lower versus higher educational attainment, living alone versus living with others, unmarried versus married, female versus male, and rural versus urban residence, respectively.

## 4 Discussion

### 4.1 Principal findings, interpretation and implications

One focus of the present study was to examine changes in the prevalence of depressive symptoms among older adults in China during and after the COVID-19 pandemic. Year-stratified pooled analyses showed an overall pattern of increase, decline and renewed increase between 2020 and 2025, with the lowest estimate observed in 2022 (16.6%) and higher estimates in the post-pandemic period. These fluctuations broadly coincide with changes in epidemic conditions and policy responses in China (115), while evidence from other settings also suggests that population mental health trajectories were not uniform across different phases of the pandemic and recovery period (116, 117). However, given the substantial between-study heterogeneity and the repeated cross-sectional nature of the evidence, these temporal patterns should be interpreted cautiously and should not be attributed to specific pandemic-related mechanisms.

More importantly, socio-demographic inequalities in depression remained broadly persistent across the pandemic and post-pandemic periods, consistent with evidence of social patterning in late-life mental health across different national settings (118, 119). The largest disparities were observed for educational attainment (OR 1.693, 95% CI 1.388–2.060), living arrangement (OR 1.671, 95% CI 1.437–1.945) and marital status (OR 1.652, 95% CI 1.467–1.860). These dimensions capture partly distinct but interconnected forms of social disadvantage. Lower educational attainment is associated with differences in socioeconomic position, health literacy and access to protective resources accumulated across the life course (6, 42, 43, 120, 121), while living alone and being unmarried may entail reduced access to day-to-day emotional, instrumental and caregiving support (9, 122–129). The persistence of these disparities despite substantial changes in the broader public health environment suggests that late-life mental health vulnerability remained strongly patterned by pre-existing social and material inequalities rather than being uniformly redistributed during the crisis.

Some variation across inequality dimensions was nevertheless observed. Marital-status-related inequality showed a small upward trend (exp(β) = 1.021), whereas the apparent attenuation in living-arrangement-related inequality was not statistically significant (exp(β) = 0.892). These divergent patterns caution against interpreting temporal variation through a single social-support mechanism. Changes in marital-status-related disparities may partly reflect loss-related vulnerability, including bereavement and widowhood (130–132), whereas living-arrangement-related differences may be more responsive to changes in social connection and community support (39, 133). Given the limited number of year-specific estimates and the exploratory nature of these analyses, however, these patterns require cautious interpretation.

Gender and urban–rural disparities further indicate that inequalities in late-life depression extend beyond educational and family domains. Older women consistently showed higher odds of depressive symptoms, in line with evidence linking gender disparities in later-life mental health to differences in caregiving burdens, adverse life experiences, social roles and cumulative resource disadvantage (134, 135). Rural older adults likewise showed higher odds than their urban counterparts, although international evidence on urban–rural disparities is less consistent (6, 136). In China, urban–rural differences in socioeconomic resources, healthcare and mental health service access, community support and opportunities for social participation may contribute to this pattern (137, 138). Evidence that such differences attenuate after adjustment for individual-, family- and community-level socioeconomic conditions further suggests that place of residence may operate partly as a spatial expression of broader structural inequalities rather than as an isolated risk factor (62).

A prominent feature of this study was the persistently high between-study heterogeneity, which remained substantial even after stratification by year, region and other subgroup dimensions. Univariable random-effects meta-regression showed that investigation year was not significantly associated with prevalence variation, whereas region and measurement tool were more closely related to between-study differences, particularly the higher estimates observed in studies using CES-D compared with PHQ. This suggests that the observed heterogeneity cannot be explained by temporal stage alone, but may reflect the combined influence of regional context, sampling frames and measurement-related variability, including differences between commonly used instruments such as the GDS-15, PHQ-9 and CES-D10, which is a common challenge in prevalence meta-analyses (33). Given the presence of genuine between-study variability, random-effects models were used to estimate mean effects under a distribution of true prevalence values (139), and emphasis was placed on the consistency and robustness of structural inequality patterns rather than on precise comparisons of absolute prevalence levels.

Taken together, the year-level fluctuation in prevalence and the relative stability of socio-demographic disparities suggest that major public health disruption may alter the overall burden of late-life depression without necessarily restructuring its underlying social distribution. This pattern is consistent with the broader literature on health inequalities (140) and echoes evidence from older adults in Hong Kong during the SARS epidemic, suggesting that the mental health consequences of major public health events may unfold within pre-existing social structures rather than simply overriding them (42). More recent international evidence during COVID-19 points in a similar direction. A scoping review of socioeconomic inequalities in depressive and anxiety symptoms found that inequalities persisted or increased in most analyses across high-income countries, despite considerable variation in their temporal trajectories (141). The present findings extend this evidence to older adults in China and across multiple dimensions of social stratification, suggesting that the persistence of mental health inequality under crisis conditions may represent a broader challenge for aging societies.

These findings also have implications for mental health preparedness during future public health emergencies. International evidence has emphasized the importance of monitoring socioeconomic inequalities over time and adapting preventive strategies to different phases of a crisis (141). For older populations, preparedness should therefore extend beyond preventing infection and monitoring average levels of psychological distress to identifying groups with persistent social vulnerability (142). Integrating routinely available socio-demographic and social-determinant information into mental health surveillance may facilitate risk stratification and more equitable allocation of preventive and supportive resources (143). In the present study, educational attainment, living arrangement, marital status, gender and place of residence emerged as readily identifiable markers of such vulnerability and may therefore help inform targeted screening, outreach and service planning. Maintaining social connection and access to community and mental health services may be particularly important for older adults with limited social or material resources, given longitudinal evidence linking social disconnectedness and perceived isolation to depressive and anxiety symptoms in later life (144). More broadly, declines in population-level prevalence should not be assumed to indicate corresponding reductions in inequality; monitoring both the overall mental health burden and its social distribution may therefore strengthen equity-oriented responses to future pandemics and other public health emergencies.

### 4.2 Strengths and limitations

This study has several strengths. First, it systematically synthesized evidence spanning both the pandemic and post-pandemic periods, allowing changes in the prevalence of depressive symptoms among older adults in China and their socio-demographic inequalities to be examined within a unified framework. Second, the included studies covered multiple provinces and both urban and rural settings across mainland China, and key dimensions of social stratification—including gender, marital status, residence, living arrangement and educational attainment—were compared using a consistent quantitative approach. Third, in addition to year-stratified pooled prevalence, we reported relative effect measures (ORs) and conducted exploratory year-level analyses based on year-specific pooled estimates, providing a more complete picture of the stability of inequality patterns under public health disruption. More broadly, evidence from China as a large and rapidly aging society offers useful contextual and comparative insight for the international literature on late-life depression inequalities.

Several limitations should also be acknowledged. First, despite acceptable overall study quality, substantial differences remained in sampling frames, survey timing, local context, and the instruments and cut-off values used to assess depressive symptoms, which probably contributed to the very high heterogeneity observed. Second, most included studies were cross-sectional, so causal inferences cannot be drawn regarding the observed associations between socio-demographic characteristics and depressive symptoms. In particular, the year-level regression of year-specific pooled ORs should be regarded as an exploratory ecological analysis, given that it was based on pooled estimates rather than study-level observations, involved a limited number of time points and may have been influenced by changes in study composition over time. Third, COVID-19 infection may be associated with depressive symptoms (145); however, information on individual infection history, frequency of infection and infection-related symptoms was not systematically available across the included studies. We were therefore unable to examine whether these infection-related characteristics were associated with depressive symptoms or contributed to the observed temporal patterns. Future studies incorporating individual-level infection and symptom data may help further distinguish infection-related mental health vulnerability from the broader social and public health consequences of the pandemic. Fourth, incomplete reporting of some stratified data may have reduced the precision and comparability of certain subgroup analyses. Fifth, although funnel plot-based tests, trim-and-fill analysis and leave-one-out sensitivity analyses did not indicate substantial instability in the main findings, selective publication or residual small-study effects cannot be fully excluded. Finally, this analysis was restricted to community-dwelling older adults in mainland China, and differences in healthcare systems, family support structures, cultural norms and public health responses may limit direct generalizability to other settings.

## 5 Conclusion

Depressive symptoms among older adults in China fluctuated across the pandemic and post-pandemic periods, while socio-demographic inequalities in depression risk remained broadly stable. Larger disparities were observed in relation to educational attainment, living arrangement and marital status, suggesting that major public health disruption did not fundamentally restructure existing inequalities in late-life depression but instead exposed and prolonged them in uneven ways. Evidence from China therefore provides a useful comparative reference for understanding how public health emergencies may shape mental health vulnerability in aging societies.

## Data Availability

The original contributions presented in the study are included in the article/Supplementary material, further inquiries can be directed to the corresponding author/s.

## References

[1] GBD 2019 Mental Disorders Collaborators. Global, regional, and national burden of 12 mental disorders in 204 countries and territories, 1990–2019: a systematic analysis for the global burden of disease study 2019. Lancet Psychiatry. (2022) 9:137–50. doi: 10.1016/S2215-0366(21)00395-3, 35026139 PMC8776563

[2] Zenebe Y Akele B W/Selassie M Necho M. Prevalence and determinants of depression among old age: a systematic review and meta-analysis. Ann General Psychiatry. (2021) 20:55. doi: 10.1186/s12991-021-00375-x, 34922595 PMC8684627

[3] Cai H Jin Y Liu R Zhang Q Su Z Ungvari GS . Global prevalence of depression in older adults: a systematic review and meta-analysis of epidemiological surveys. Asian J Psychiatry. (2023) 80:103417. doi: 10.1016/j.ajp.2022.103417 36587492

[4] Huang Y Wang Y Wang H Liu Z Yu X Yan J . Prevalence of mental disorders in China: a cross-sectional epidemiological study. Lancet Psychiatry. (2019) 6:211–24. doi: 10.1016/S2215-0366(18)30511-X, 30792114

[5] Chen YJ Li Y. Wang H. Ding H. [The impact of anxiety and depression on sleep quality in rural older adults in China: the mediating role of social support]. Journal of Nanjing Medical University (Social Sciences) (2025) 25:36–41. doi: 10.7655/NYDXBSSS240387 [in Chinese].

[6] Li Y Chen C Tan H Chen W Feng S Ma Y . Prevalence and risk factors for depression in older people in xi′an China: a community-based study. Int J Geriatr Psychiatry. (2012) 27:31–9. doi: 10.1002/gps.2685 21284042

[7] Wang J Wang Y Chen S Fu T Sun G. Urban-rural differences in key factors of depressive symptoms among Chinese older adults based on random forest model. J Affect Disord. (2024) 344:292–300. doi: 10.1016/j.jad.2023.10.017, 37820963

[8] Yan XY Huang SM Huang CQ Wu WH Qin Y. Marital status and risk for late life depression: a meta-analysis of the published literature. J Int Med Res. (2011) 39:1142–54. doi: 10.1177/147323001103900402, 21986116

[9] Wang Q Song D Lin Q Tao A Zhang Y Li X . The critical role of physical frailty and function on depressive symptoms among community-dwelling older adults in China: a cross-sectional study. Front Psych. (2023) 14:1134427. doi: 10.3389/fpsyt.2023.1134427, 36873198 PMC9975574

[10] Gostynski M Ajdacic-Gross V Gutzwiller F Michel JP Herrmann F. Depression bei Betagten in der Schweiz. Nervenarzt. (2002) 73:851–60. doi: 10.1007/s00115-002-1369-1, 12215876

[11] Phelan JC Link BG Tehranifar P. Social conditions as fundamental causes of health inequalities: theory, evidence, and policy implications. J Health Soc Behav. (2010) 51:S28–40. doi: 10.1177/0022146510383498, 20943581

[12] Mohd TAMT Yunus RM Hairi F Hairi NN Choo WY. Social support and depression among community dwelling older adults in Asia: a systematic review. BMJ Open. (2019) 9:e026667. doi: 10.1136/bmjopen-2018-026667 PMC6661578 31320348

[13] Albert PR. Why is depression more prevalent in women? J Psychiatry Neurosci. (2015) 40:219–21. doi: 10.1503/jpn.150205, 26107348 PMC4478054

[14] Purtle J Nelson KL Yang Y Langellier B Stankov I Diez Roux AV. Urban–rural differences in older adult depression: a systematic review and meta-analysis of comparative studies. Am J Prev Med. (2019) 56:603–13. doi: 10.1016/j.amepre.2018.11.008, 30777704

[15] Corneliusson L Gustafson Y Olofsson B. Prevalence of depressive disorders among the very old in the 21st century. J Affect Disord. (2024) 362:706–15. doi: 10.1016/j.jad.2024.07.062, 39029671

[16] Lu L Shen H Tan L Huang Q Chen Q Liang M . Prevalence and factors associated with anxiety and depression among community-dwelling older adults in Hunan, China: a cross-sectional study. BMC Psychiatry. (2023) 23:107. doi: 10.1186/s12888-023-04583-5, 36793025 PMC9930706

[17] Bambra C Riordan R Ford J Matthews F. The COVID-19 pandemic and health inequalities. J Epidemiol Community Health. (2020) 74:964–8. doi: 10.1136/jech-2020-214401, 32535550 PMC7298201

[18] World Health Organization WHO timeline: COVID-19 (2020). Available online at: https://www.who.int/news/item/27-04-2020-who-timeline-covid-19 (Accessed March 17, 2026).

[19] Ministry of Civil Affairs of the People’s Republic of China Statistical bulletin on the development of civil affairs in 2023. (2024). Available online at: https://www.mca.gov.cn/ (Accessed March 10, 2026).

[20] Zhao X Du X Bai S Zheng P Zhou X Wang Z. Differences in depression prevalence among older adults in China before and during the COVID-19 pandemic: a systematic review and meta-analysis. PeerJ Comput Sci. (2025) 11:e19251. doi: 10.7717/peerj.19251 PMC11995894 40231071

[21] National Bureau of Statistics of China. Statistical communiqué of the People’s Republic of China on the 2024 national economic and social Development. Beijing, China: National Bureau of Statistics of China (2025).

[22] Han Y He Y Lyu J Yu C Bian M Lee L. Aging in China: perspectives on public health. Glob Health J. (2020) 4:11–7. doi: 10.1016/j.glohj.2020.01.002

[23] Wu Y Cao Z Yang J Bi X Xiong W Feng X . Innovative public strategies in response to COVID-19: a review of practices from China. Health Care Sci. (2024) 3:383–408. doi: 10.1002/hcs2.122, 39735280 PMC11671218

[24] Wang Y Luo B Wang J Liao S. The psychological impact of the COVID-19 pandemic in the elderly in Southwest China: a longitudinal study based on generalized estimating equations. Int J Disaster Risk Reduct. (2023) 88:103609. doi: 10.1016/j.ijdrr.2023.103609, 36843888 PMC9942486

[25] Zhang S Dong S Yu Z Bi S Wen W Li J. Internet usage, social participation, and depression symptoms among middle-aged and older adult Chinese individuals before and during the COVID-19 pandemic: evidence from the China health and retirement longitudinal observational study. J Med Internet Res. (2025) 27:e67039. doi: 10.2196/67039, 41024412 PMC12479384

[26] Amir-Behghadami M Janati A. Population, intervention, comparison, outcomes and study (PICOS) design as a framework to formulate eligibility criteria in systematic reviews. Emerg Med J. (2020) 37:387–7. doi: 10.1136/emermed-2020-209567, 32253195

[27] Page MJ McKenzie JE Bossuyt PA Boutron I Hoffmann TC Mulrow CD . The PRISMA 2020 statement: an updated guideline for reporting systematic reviews. J Clin Epidemiol. (2021) 134:178–89. doi: 10.1136/bmj.n71 33789819

[28] Rostom A Dubé C Cranney A Saloojee N Sy R Garritty C . Celiac Disease: Summary. Evidence Report/Technology Assessment No. 104. Rockville, MD: Agency for Healthcare Research and Quality (2004).

[29] Zeng X Zhang Y Kwong JSW Zhang C Li S Sun F . The methodological quality assessment tools for preclinical and clinical studies, systematic review and meta-analysis, and clinical practice guideline: a systematic review. J Evid Based Med. (2015) 8:2–10. doi: 10.1111/jebm.12141, 25594108

[30] Kanters S. "Fixed- and random-effects models". In: Evangelou E Veroniki AA, editors. Meta-Research: Methods and Protocols. New York, NY: Springer US (2021). p. 41–65.

[31] Higgins JPT Thompson SG. Quantifying heterogeneity in a meta-analysis. Stat Med. (2002) 21:1539–58. doi: 10.1002/sim.1186, 12111919

[32] Migliavaca CB Stein C Colpani V Barker TH Ziegelmann PK Munn Z . Meta-analysis of prevalence: I 2 statistic and how to deal with heterogeneity. Res Synth Methods. (2022) 13:363–7. doi: 10.1002/jrsm.1547 35088937

[33] Deeks JJ Higgins JPT Altman DG McKenzie JE Veroniki AA. (eds.)."Chapter 10: Analysing data and undertaking meta-analyses". In: Higgins JPT Thomas J Chandler J Cumpston M Li T Page MJ , editors. Cochrane Handbook for Systematic Reviews of Interventions version 6.5. Cochrane (2024) Available online at: https://training.cochrane.org/handbook/current/chapter-10 (Accessed April 20, 2026).

[34] Lorant V Deliege D Eaton W Robert A Philippot P Ansseau M. Socioeconomic inequalities in depression: a meta-analysis. Am J Epidemiol. (2003) 157:98–112. doi: 10.1093/aje/kwf182, 12522017

[35] McKenzie JE Veroniki AA. A brief note on the random-effects meta-analysis model and its relationship to other models. J Clin Epidemiol. (2024) 174:111492. doi: 10.1016/j.jclinepi.2024.111492 39098563

[36] Altman DG Bland JM. How to obtain the P value from a confidence interval. BMJ. (2011) 343:d2304. doi: 10.1136/bmj.d2304, 22803193

[37] UCLA Institute for Digital Research and Education. FAQ: How do I interpret odds ratios in logistic regression? Available online at: https://stats.oarc.ucla.edu/other/mult-pkg/faq/general/faq-how-do-i-interpret-odds-ratios-in-logistic-regression/ (Accessed April 20, 2026).

[38] Chen BY Mo Y Chen LJ Zhang ZJ Wang FL Cai L. Prevalence of depression and its relationship with social support among the Bai ethnic minority elderly in rural Yunnan Province. Chin Rural Health Serv Adm. (2023) 43:141–6. doi: 10.19955/j.cnki.1005-5916.2023.02.012 (in Chinese)

[39] Qin ZL Deng SS Li C. Analysis of depression status and its influence on the quality of life of the elderly in western Guangxi. J Youjiang Med Univ Natl. (2022) 44:881–6. (in Chinese)

[40] Yu JQ Wu YJ Zhang Y He YZ Cai YT Zheng JZ . Correlation between depressive symptoms and health promotion behavior in the elderly: based on a structural equation model. Chin J Dis Control Prev. (2022) 26:1072–7. doi: 10.16462/j.cnki.zhjbkz.2022.09.014 (in Chinese)

[41] Qin YW Li HJ Ying X Tian FY Li SB Wan LP . Analysis on status and influencing factors of depressive symptoms among the elderly aged 65 years and above in rural areas, Linyi city. Prev Med Trib. (2022) 28:161–5. doi: 10.16406/j.pmt.issn.1672-9153.2022.03.013 (in Chinese)

[42] Liu JX Luo JS Ji SQ Wang C Zeng ML Li YL . Depression and its influencing factors in rural poor elderly in western Hunan province. J Community Med. (2022) 20:1243–8. doi: 10.19790/j.cnki.JCM.2022.22.01 (in Chinese)

[43] Cui CH Dong A Yang Q. Mediating effect of leisure activities on cognitive frailty and depression in community-dwelling elderly. Chin J Mult Organ Dis Elderly. (2022) 21:45–8.doi: 10.11915/j.issn.1671-5403.2022.01.010 (in Chinese)

[44] Xu K. Impact of COVID-19 cognition on depression among older adults during the normalization of epidemic prevention and control: the chain mediating effect of discrimination perception and risk perception. China J Health Psychol. (2022) 30:1496–501. doi: 10.13342/j.cnki.cjhp.2022.10.011 (in Chinese)

[45] Liu CC Chen LQ Xie BQ Chen Y Wang JL Xu HQ. Prevalence and determinants of social isolation among community-living oldest-old adults. J Nurs Sci. (2022) 37:98–102. (in Chinese)

[46] Lu C Guan T Yang J Zhang DM Lin XH. Residence patterns, level of community service and depressive symptoms of older adults in Shenzhen. Scientific Research on Aging. (2022) 10:44–57. doi: 10.3969/j.issn.2095-5898.2022.05.005 (in Chinese)

[47] Hao R Jin H Zuo J Wu Y Sun X Hu J. The multiple mediating effect of family health and perceived social support on depressive symptoms in older adults: a cross-sectional national survey in China. J Affect Disord. (2023) 327:348–54. doi: 10.1016/j.jad.2023.01.097 36731543

[48] Li Y Lin Z Wu Y. Exploring depression among the elderly during the COVID-19 pandemic: the effects of the big five, media use, and perceived social support. Int J Environ Res Public Health. (2022) 19:13534. doi: 10.3390/ijerph192013534, 36294113 PMC9602753

[49] Li R Zong ZY Gu XX Wang DN Dong C Sun C . Higher dietary diversity as a protective factor against depression among older adults in China: a cross-sectional study. Ann Palliat Med. (2022) 11:1278–89. doi: 10.21037/apm-21-1449, 34775772

[50] Li S Guo B Lu X Yang Q Zhu H Ji Y . Investigation of mental health literacy and status of residents during the re-outbreak of COVID-19 in China. Front Public Health. (2022) 10:895553. doi: 10.3389/fpubh.2022.895553, 35903390 PMC9315293

[51] Ding KR Wang SB Xu WQ Lin LH Liao DD Chen HB . Low mental health literacy and its association with depression, anxiety and poor sleep quality in Chinese elderly. Asia Pac Psychiatry. (2022) 14:e12520. doi: 10.1111/appy.12520, 36210054

[52] Hou P Xue H Zhang Y Ping Y Zheng Y Wang Y . Mediating effect of loneliness in the relationship between depressive symptoms and cognitive frailty in community-dwelling older adults. Brain Sci. (2022) 12:1341. doi: 10.3390/brainsci12101341, 36291275 PMC9599707

[53] Mao S Lu N Xiao C. Perceived neighborhood environment and depressive symptoms among older adults living in urban China: the mediator role of social capital. Health Soc Care Community. (2022) 30:e1977–90. doi: 10.1111/hsc.13631 34725894

[54] Liu W Yu F Geldsetzer P Yang J Wang Z Golden T . Prevalence of depression in China during the early stage of the COVID-19 pandemic: a cross-sectional study in an online survey sample. BMJ Open. (2022) 12:e056667. doi: 10.1136/bmjopen-2021-056667, 35264364 PMC8914405

[55] Wang S Zhang Y Guan Y Ding W Meng Y Hu H . A nationwide evaluation of the prevalence of and risk factors associated with anxiety, depression and insomnia symptoms during the return-to-work period of coronavirus disease 2019 in China. Soc Psychiatry Psychiatr Epidemiol. (2021) 56:2275–86. doi: 10.1007/s00127-021-02046-4, 33616693 PMC7898251

[56] Liu A Peng Y Zhu W Zhang Y Ge S Zhou Y . Analysis of factors associated with depression in community-dwelling older adults in Wuhan, China. Front Aging Neurosci. (2021) 13:743193. doi: 10.3389/fnagi.2021.743193, 34867276 PMC8636125

[57] Zhou R Chen H Zhu L Chen Y Chen B Li Y . Mental health status of the elderly Chinese population during COVID-19: an online cross-sectional study. Front Psych. (2021) 12:645938. doi: 10.3389/fpsyt.2021.645938, 34054603 PMC8149938

[58] Liang W Duan Y Shang B Hu C Baker JS Lin Z . Precautionary behavior and depression in older adults during the COVID-19 pandemic: an online cross-sectional study in Hubei, China. Int J Environ Res Public Health. (2021) 18:1853. doi: 10.3390/ijerph18041853, 33672885 PMC7918441

[59] Wang GY Tang SF. Perceived psychosocial health and its socio-demographic correlates in times of the COVID-19 pandemic: a community-based online study in China. Infect Dis Poverty. (2020) 9:148–68. doi: 10.1186/s40249-020-00770-8, 33106187 PMC7586696

[60] Wang S Zhang Y Ding W Meng Y Hu H Liu Z . Psychological distress and sleep problems when people are under interpersonal isolation during an epidemic: a nationwide multicenter cross-sectional study. Eur Psychiatry. (2020) 63:e77. doi: 10.1192/j.eurpsy.2020.78, 32854786 PMC7503168

[61] He L Wang J Wang F Zhang L Liu Y Xu F. Depression symptoms and quality of life in empty-nest elderly among Chengdu: a cross-sectional study. Front Psych. (2022) 13:1003261. doi: 10.3389/fpsyt.2022.1003261, 36424995 PMC9679215

[62] Li H Pang M Wang J Xu J Kong F. Effects of health service utilization and informal social support on depression, anxiety, and stress among the internal migrant elderly following children in Weifang, China. Int J Environ Res Public Health. (2022) 19:14640. doi: 10.3390/ijerph192214640, 36429375 PMC9691240

[63] Yu HD Pan MM Gu HQ Liu T Dai J Liu LH . Correlation analysis between depressive symptoms, cognitive status and nutritional metabolism among community-dwelling older adults. Chin Gen Pract. (2024) 27:3540–5. doi: 10.12114/j.issn.1007-9572.2023.0591 (in Chinese)

[64] Zhao ZH Wang LY Su S Li WX. Correlation analysis between cognitive function and depression and anxiety symptoms in community-dwelling older adults. Guoji Jingshenbingxue Zazhi. (2024) 51:1154–60. doi: 10.13479/j.cnki.jip.2024.04.005 (in Chinese)

[65] Liu M Wang XY Duan Y Gao XF. [Study on the prevalence and influencing factors of depression among older adults in rural areas of Sichuan Province]. Mod Prev Med. (2024) 51:2892–2897, 2910. doi: 10.20043/j.cnki.MPM.202310259 (in Chinese)

[66] Zong LX Ge MH. Establishment and validation of a multifactor risk prediction model for depression among elderly residents in Weifang. J Psychiatry. (2022) 35:529–34. doi: 10.3969/j.issn.2095-9346.2023.05.017 (in Chinese)

[67] Zhang Y Jing Q Guo TT Zhao XQ Dong ZW Ji LH . Analysis of the mediating effect of daily living ability on the relationship between sleep duration and depressive symptoms in the elderly. Mod Prev Med. (2024) 51:3146–3150,3156. doi: 10.20043/j.cnki.MPM.202405449 (in Chinese)

[68] Liu YT Chen FH Yan P Zhang XH Li H Yang Y . Mediating effect of social support on anxiety, depression and quality of life in older adults. Int J Geriatr. (2023) 44:330–3. doi: 10.3969/j.issn.1674-7593.2023.03.016 (in Chinese)

[69] Yang L Guo D Wang J Guo YT Li ZY. Prevalence and determinants of depression among older adults relocated for poverty alleviation in Shanxi Province: a cross-sectional survey. Chin J Public Health. (2024) 40:686–90. doi: 10.11847/zgggws1143229 (in Chinese)

[70] Liu SB Li XY Sun GH. The status of anxiety and depression and their influencing factors among older adults in a county community. Guoji Jingshenbingxue Zazhi. (2024) 51:788–91. doi: 10.13479/j.cnki.jip.2024.03.039 (in Chinese)

[71] Huang W Qiu ZJ. Survey on the current status of depression among elderly residents in Hunan Province and construction of a risk prediction model. J Yueyang Voc Tech Coll. (2024) 39:71–8. doi: 10.13947/j.cnki.yyzyxb.2024.01.012 (in Chinese)

[72] Zhao QP Cui Y Zhang XY Zheng M Liu SS Wang FL. Relationship of resilience and family intergenerational solidarity with frailty and depression among middle-aged and older adults. J Nurses Train. (2023) 38:1272–5. doi: 10.16821/j.cnki.hsjx.2023.14.005 (in Chinese)

[73] Li Q Li GH Liu L Liu DL Yu ZZ Zuo M . Study on the relationship between nicotine dependence and depression among rural Dai older adults in Jinggu County, Yunnan Province. Mod Prev Med. (2024) 51:854–8. doi: 10.20043/j.cnki.MPM.202310135 (in Chinese)

[74] Su HX Li M Li YW Xie MH Li SQ Liang B. A survey of anxiety and depression among older adults in a community of Bengbu city and analysis of influencing factors. Chin J Clin Healthc. (2023) 26:787–91. doi: 10.3969/j.issn.1672-6790.2023.06.016 (in Chinese)

[75] Lin YM Feng Y Xue WC Cao WX. A survey on anxiety and depression among elderly people in rural areas along the Yellow River in Lüliang City, Shanxi Province. Jiankang Jiaoyu Yu Jiankang Cujin. (2023) 18:584–7. doi: 10.16117/j.cnki.31-1974/r.202306584 (in Chinese)

[76] Yang J Lv XZ Shang L Li HZ Lu C Zhang M . Detection rates and related factors of depression and anxiety among older adults in Shenzhen. Chin Ment Health J. (2023) 37:373–9. doi: 10.3969/j.issn.1000-6729.2023.05.003 (in Chinese)

[77] Ge HJ Dong SH Liu Y Qi YT Du J Gao J . Identification of depression-related factors among older adults in China based on social determinants of health. Mod Prev Med. (2023) 50:4147–4152,4163. doi: 10.20043/j.cnki.MPM.202305277 (in Chinese)

[78] Jiang Y Lu Y Cui J Chu A. 24-hour movement behaviors time allocation and depression among Chinese community-dwelling older adults: a latent profile analysis. Geriatr Nurs. (2024) 58:382–7. doi: 10.1016/j.gerinurse.2024.05.007, 38880078

[79] Zhang Y Yu G Bai W Wu S Geng X Zhang W . Association of depression and sleep quality with frailty: a cross-sectional study in China. Front Public Health. (2024) 12:1361745. doi: 10.3389/fpubh.2024.1361745, 38645453 PMC11026860

[80] Hu Q Song Y Wang S Lin L Ke Y Zhang P. Association of subjective cognitive complaints with poor sleep quality: a cross-sectional study among Chinese elderly. Int J Geriatr Psychiatry. (2023) 38:e5956. doi: 10.1002/gps.5956, 37329227

[81] Gan S Yao X Li Z. Correlation between social network and mental health in family-oriented older migrants: a study in two cities in China. Aging Health Res. (2024) 4:100201. doi: 10.1016/j.ahr.2024.100201

[82] Xiong H Ma F Tang D Liu D. Correlations among nicotine dependence, health-related quality of life, and depression in current smokers: a cross-sectional study with a mediation model. Front Psych. (2024) 15:1455918. doi: 10.3389/fpsyt.2024.1455918, 39257561 PMC11384568

[83] Peng X Zhang S You L Hu W Jin S Wang J. Prevalence and correlates of depression and anxiety symptoms among older adults in Shenzhen, China: a cross-sectional population-based study. BMJ Open. (2024) 14:e077078. doi: 10.1136/bmjopen-2023-077078, 38341212 PMC10862290

[84] He ZF Tan WY Ma H Shuai Y Shan Z Zhai J . Prevalence and factors associated with depression and anxiety among older adults: a large-scale cross-sectional study in China. J Affect Disord. (2024) 346:135–43. doi: 10.1016/j.jad.2023.11.022, 37949242

[85] Zeng H Zhao W Wang R Li H Wang L Zeng X. Association between swallowing function and depressive symptoms among community-dwelling older adults: a cross-sectional study in Central China. J Affect Disord. (2025) 380:78–86. doi: 10.1016/j.jad.2025.03.113, 40120954

[86] Wang X Zhu B Li JP Li X Zhang L Wu Y . The moderating effect of frailty on the network of depression, anxiety, and loneliness in community-dwelling older adults. J Affect Disord. (2025) 375:508–16. doi: 10.1016/j.jad.2025.01.118, 39862977

[87] Wu J Xu H Qin ZZ Wang CC Zhang Y Hong X. The effects of physical activity, depressive symptoms and their interaction on cognitive function among older adults in Nanjing. Chin J Prev Control Chronic Dis. (2025) 33:29–32. doi: 10.16386/j.cjpccd.issn.1004-6194.20240705.0485 (in Chinese)

[88] Zhang YX Zhang SM Jiang SQ Chen SR Li J Zhou CC. The relationship between adverse childhood experiences and depressive symptoms among rural older adults in Shandong Province. Med Soc. (2025) 38:130–6. doi: 10.13723/j.yxysh.2025.03.018 (in Chinese)

[89] Yang T Liu Y Li X Gai Y Xie Z Wang J . Association between household solid fuel use for cooking and depressive symptoms among middle-aged and elderly adults in rural China: evidence from the China family panel studies database. J Environ Occup Med. (2025) 42:926–31. doi: 10.5555/20250457080

[90] Zhou L Zhang A Liu Q Ji Q Ye M Xu Y . Association between religious service attendance and depression, anxiety among older Amdo Tibetans. Int J Geriatr Psychiatry. (2025) 40:e70076. doi: 10.1002/gps.70076, 40229985

[91] Du S Lyu L Cai Z Wu Y Zhou H. Association between sleep quality and depressive symptoms in community-dwelling older adults: the mediating role of loneliness. J Sichuan Univ Med Ed. (2025) 56:458. doi: 10.12182/20250360605 PMC12207046 40599276

[92] Liu D Yu Z Liu L Li G Li X Ruan C . Socioeconomic disparities in the prevalence of depression and anxiety, and their associations with diabetes in rural Southwest China. BMC Public Health. (2025) 25:668. doi: 10.1186/s12889-025-21837-x, 39966812 PMC11837715

[93] Tian W Yan G Zhang X Shui D Liu T Yang J . The prevalence, risk factors, awareness and treatment of depression and anxiety among adults aged 30 years and older in rural China: a cross-sectional study. J Affect Disord. (2025) 387:119516. doi: 10.1016/j.jad.2025.119516, 40441637

[94] Wei SS Liu KR Wang RX Tao J He B Wang YH. Relationship of osteosarcopenia with depressive symptoms and physical activity in community-dwelling older adults. Chin Ment Health J. (2025) 39:611–6. doi: 10.3969/j.issn.1000-6729.2025.07.06 (in Chinese)

[95] Liu SX Qi L Chen C Tan C Qian H Dai XL . Associations of lean body mass, fat mass and their indices with depressive symptoms among adults aged 65 years and above in 18 longevity areas of China. Natl Med J China. (2025) 105:1840–6. doi: 10.3760/cma.j.cn112137-20250212-00318 (in Chinese) 40484846

[96] Liu CM Liu AN Liu MC. Effects of intergenerational support and depression on social isolation in older adults. Chin Nurs Res. (2025) 39:1267–71. doi: 10.12102/j.issn.1009-6493.2025.08.006 (in Chinese)

[97] Li D Zuo PX Duan YL Yin FF Cao YW. Effects of social isolation and depression on cognitive function in community-dwelling older adults. J Nongken Med. (2025) 47:159–64. doi: 10.27332/d.cnki.gshzu.2024.000329 (in Chinese)

[98] Zhao HR Xiang XL Wang ZJ Xia Y Ma M Wang HD . Impact of social activities on quality of life among older adults: the chain mediating effect of depression and life satisfaction. Mod Prev Med. (2025) 52:1860–6. doi: 10.20043/j.cnki.MPM.202412498 (in Chinese)

[99] Feng W Li ZX Zhang W Yuan J He L Li LJ . A study on the current status and influencing factors of cognitive decline and depressive state among the elderly in Karamay City. Linchuang Yixue Jinzhan. (2025) 15:1350–7. doi: 10.12677/acm.2025.1572134 (in Chinese)

[100] Lu J Zhang F Bao LM Yang TL Lu HL. The effect of sedentary behavior on the incidence of depression among community-dwelling older adults in Ankang, Shaanxi. Pract Prev Med. (2025) 32:1126–9. doi: 10.3969/j.issn.1006-3110.2025.09.022 (in Chinese)

[101] Chen RY Li R Li XW Jiang ZD Li YJ Liu WL . Analysis on status and influencing factors of depressive symptoms among the elderly aged 60 years and above in Shandong Province. Prev Med Trib. (2025) 31:433–7. doi: 10.16406/j.pmt.issn.1672-9153.2025.6.07 (in Chinese)

[102] Dong P Wu H Chai Y Huang P Huang D Lai L . Impact of multimorbidity and cognitive impairment on depressive symptoms in community-dwelling older adults and their interaction effects. Sci Rep. (2025) 15:15033. doi: 10.1038/s41598-025-99547-y, 40301660 PMC12041597

[103] Dong P Zhang X Yin W Shi Y Xu M Li H . Relationship between short video addiction tendency and depression among rural older adults: cross-sectional study. J Med Internet Res. (2025) 27:e75938. doi: 10.2196/75938, 40587851 PMC12278998

[104] Wu S Yang L Mo H Li L Li Z Ying Y. Prevalence and factors associated with anxiety and depression among the older people in ethnic minority areas in southern China: a cross-sectional study. Front Public Health. (2025) 13:1494629. doi: 10.3389/fpubh.2025.1494629, 40630414 PMC12234299

[105] Zhang XF Yu LL Zhang GH Ma J Qi C Chen LX. Depression status and its influencing factors among the population aged 65 years and above in Shandong Province. Chin J Dis Control Prev. (2025) 29:1388–93. doi: 10.16462/j.cnki.zhjbkz.2025.12.004 (in Chinese)

[106] Lan Y Yang JY Shama SJ Jiang Q Qin JT Wu Q . Study on the correlation between health-promoting lifestyles and depressive tendencies among older adults of Yi ethnic group in rural areas. Soft Science of Health. (2025) 39:45–50. doi: 10.3969/j.issn.1003-2800.2025.10.008 (in Chinese)

[107] Wang YY Bian YH Gao B. Actor-partner interdependence model analysis of the relationship between individual social capital and depressive symptoms among older couples across different socioeconomic statuses. Journal of Sichuan University (Medical Sciences). (2025) 56:1344–50. doi: 10.12182/20250960109 (in Chinese) PMC12709098 41416151

[108] Jing YR Wang XN Zhan GL Zhang YS Li XX Wang Y. Research on the association between socioeconomic status and mild cognitive impairment in community-dwelling older adults in a district of Shanghai. Med Soc. (2025) 38:27–33. doi: 10.13723/j.yxysh.2025.09.004 (in Chinese)

[109] Huang YY Wang DH Mao CX Yi Y Huang GY Jiang SH . Study of the effect of self-perceived hearing status on depression in middle-aged and older people in the community. Chinese Journal of Otorhinolaryngology Head and Neck Surgery. (2025) 60:1154–62. doi: 10.3760/cma.j.cn115330-20241027-00602 (in Chinese), 41073294

[110] Tian YH Liu LW Yang C Ling C Yang XX Fan HJ . Associations and sex differences between depression and cognitive function among urban older adults. Chin Gen Pract. (2025) 28:47–52. doi: 10.12114/j.issn.1007-9572.2023.0642 (in Chinese)

[111] Lin X Huang LF Chen Z Liu YF Gong FH. A study on the correlation between depressive symptoms and lifestyles among rural older adults in Hunan Province. Journal of Hunan Normal University (Medical Science). (2025) 3:198–204. doi: 10.3969/j.issn.1673-016X.2025.03.034 (in Chinese)

[112] Dong M Wang SB Tan WY He HB Hou CL. Network analysis of depressive, anxiety, insomnia symptoms and cognitive function among community-dwelling older adults in Guangdong Province. Chin J Psychiatry. (2026) 59:22–9. doi: 10.3760/cma.j.cn113661-20250425-00190 (in Chinese)

[113] Zhang JY Zhou ST Li SF Chen YM Jia ZY Wang YY . Effect of depressive symptoms on subjective well-being among younger older adults: the moderating role of subjective income level. J Humanit Soc Sci. (2026) 2:142–8. doi: 10.70693/rwsk.v2i4.402 (in Chinese)

[114] Xu N Ma X Hu Q Du J He J Wang W . Association between multimorbidity and childhood socioeconomic status with depressive symptoms among middle-aged and older adults in rural western China. Sci Rep. (2026) 16:6600. doi: 10.1038/s41598-026-37666-w, 41611932 PMC12914049

[115] Van Winkle Z Ferragina E Recchi E. The unexpected decline in feelings of depression among adults ages 50 and older in 11 European countries amid the COVID-19 pandemic. Socius. (2021) 7:23780231211032741. doi: 10.1177/23780231211032741

[116] van den Besselaar JH Vroomen JLM Buurman BM Hertogh CMPM Huisman M Kok AAL . Symptoms of depression, anxiety, and perceived mastery in older adults before and during the COVID-19 pandemic: results from the longitudinal aging study Amsterdam. J Psychosom Res. (2021) 151:110656. doi: 10.1016/j.jpsychores.2021.110656, 34741872 PMC8556729

[117] Li H He X Hu TY Xie X. Past lesson works: SARS memory moderates the relationship between media use and protective behavior during COVID-19 pandemic in China. J Health Psychol. (2023) 28:203–15. doi: 10.1177/13591053221105349, 35733346 PMC9982424

[118] Silva C Fonseca C Ferreira R Weidner A Morgado B Lopes MJ . Depression in older adults during the COVID-19 pandemic: a systematic review. J Am Geriatr Soc. (2023) 71:2308–25. doi: 10.1111/jgs.18363, 37029710

[119] Noguchi T Hayashi T Kubo Y Tomiyama N Ochi A Hayashi H. Living alone and depressive symptoms among older adults in the COVID-19 pandemic: role of non-face-to-face social interactions. J Am Med Dir Assoc. (2023) 24:17–21.e4. doi: 10.1016/j.jamda.2022.10.014, 36435273 PMC9613798

[120] Long C Yang W Glaser K. Social support, cognition, and mental health among older people in China: a longitudinal life course study. Soc Sci Med. (2025) 381:118279. doi: 10.1016/j.socscimed.2025.118279 40479799

[121] Sinkewicz M Rostant O Zivin K McCammon R Clarke P. A life course view on depression: social determinants of depressive symptom trajectories over 25 years of Americans’ changing lives. SSM Popul Health. (2022) 18:101125. doi: 10.1016/j.ssmph.2022.101125, 35664926 PMC9160836

[122] McKinnon B Harper S Moore S. The relationship of living arrangements and depressive symptoms among older adults in sub-Saharan Africa. BMC Public Health. (2013) 13:682. doi: 10.1186/1471-2458-13-682, 23886221 PMC3737026

[123] Xu R Liu Y Mu T Ye Y Xu C. Determining the association between different living arrangements and depressive symptoms among over-65-year-old people: the moderating role of outdoor activities. Front Public Health. (2022) 10:954416. doi: 10.3389/fpubh.2022.954416, 35991056 PMC9386358

[124] Zhang Y Liu Z Zhang L Zhu P Wang X Huang Y. Association of living arrangements with depressive symptoms among older adults in China: a cross-sectional study. BMC Public Health. (2019) 19:1017. doi: 10.1186/s12889-019-7350-8, 31357982 PMC6664592

[125] Zheng G Zhou B Fang Z Jing C Zhu S Liu M . Living alone and the risk of depressive symptoms: a cross-sectional and cohort analysis based on the China health and retirement longitudinal study. BMC Psychiatry. (2023) 23:853. doi: 10.1186/s12888-023-05370-y, 37978367 PMC10655346

[126] Luo F Guo L Thapa A Yu B. Social isolation and depression onset among middle-aged and older adults in China: moderating effects of education and gender differences. J Affect Disord. (2021) 283:71–6. doi: 10.1016/j.jad.2021.01.022, 33524661

[127] Chen Y. Risk factors for depression among older adults living alone in Shanghai, China. Psychogeriatrics. (2022) 22:780–5. doi: 10.1111/psyg.12882, 36319268

[128] Curran E Rosato M Ferry F Leavey G. Prevalence and factors associated with anxiety and depression in older adults: gender differences in psychosocial indicators. J Affect Disord. (2020) 267:114–22. doi: 10.1016/j.jad.2020.02.018, 32063562

[129] Karaköse S. Marital satisfaction and depression in older adults: a literature review. J Aging Long-Term Care. (2022) 5:7–16. doi: 10.51819/jaltc.2022.1127139

[130] Zaninotto P Iob E Di Gessa G Steptoe A. Recovery of psychological wellbeing following the COVID-19 pandemic: a longitudinal analysis of the English longitudinal study of ageing. Aging Ment Health. (2025) 29:651–9. doi: 10.1080/13607863.2025.2450260, 39933557 PMC11957934

[131] Wang H Verdery AM Margolis R Smith-Greenaway E. Bereavement from COVID-19, gender, and reports of depression among older adults in Europe. J Gerontol B Psychol Sci Soc Sci. (2022) 77:e142–9. doi: 10.1093/geronb/gbab132, 34252179 PMC8411377

[132] Collins T Allen S Scarbrough-Jelfs C Klidzia S Lai F Wilson-Menzfeld G. “You kind of want the world to stop, and it did”: shared experiences of widowhood during and following Covid-19. Death Stud. (2025):1–19. doi: 10.1080/07481187.2025.2603969, 41412117

[133] Lau ALD Chi I Cummins RA Lee TMC Chou KL Chung LWM. The SARS (severe acute respiratory syndrome) pandemic in Hong Kong: effects on the subjective wellbeing of elderly and younger people. Aging Ment Health. (2008) 12:746–60. doi: 10.1080/13607860802380607, 19023726

[134] Luppa M Sikorski C Luck T Ehreke L Konnopka A Wiese B . Age- and gender-specific prevalence of depression in latest-life-systematic review and meta-analysis. J Affect Disord. (2012) 136:212–21. doi: 10.1016/j.jad.2010.11.033, 21194754

[135] Piccinelli M Wilkinson G. Gender differences in depression: critical review. Br J Psychiatry. (2000) 177:486–92. doi: 10.1192/bjp.177.6.486, 11102321

[136] Kong X Xiao Q Li J. Urban-rural comparison of factors related to depressive symptoms in the elderly. Chin Ment Health J. (2018) 32:648–55. doi: 10.3969/j.issn.1000-6729.2018.08.005

[137] Chow N Bai X. Modernization and its impact on Chinese older people’s perception of their own image and status. Int Soc Work. (2011) 54:800–15. doi: 10.1177/0020872811406

[138] Li LW Liu J Xu H Zhang Z. Understanding rural-urban differences in depressive symptoms among older adults in China. J Aging Health. (2016) 28:341–62. doi: 10.1177/0898264315591003, 26100620 PMC4893784

[139] Barendregt JJ Doi SA Lee YY Norman RE Vos T. Meta-analysis of prevalence. J Epidemiol Community Health. (2013) 67:974–8. doi: 10.1136/jech-2013-203104, 23963506

[140] Link BG Phelan J. Social conditions as fundamental causes of disease. J Health Soc Behav. (1995) 36:80–94. doi: 10.2307/2626958 7560851

[141] Herrmann K Beese F Wollgast L Mauz E Kersjes C Hoebel J . Temporal dynamics of socioeconomic inequalities in depressive and anxiety symptoms during the COVID-19 pandemic: a scoping review. Front Public Health. (2024) 12:1397392. doi: 10.3389/fpubh.2024.1397392, 39022423 PMC11252079

[142] Moreno C Wykes T Galderisi S Nordentoft M Crossley N Jones N . How mental health care should change as a consequence of the COVID-19 pandemic. Lancet Psychiatry. (2020) 7:813–24. doi: 10.1016/S2215-0366(20)30307-2, 32682460 PMC7365642

[143] Ganatra S Khadke S Kumar A Khan S Javed Z Nasir K . Standardizing social determinants of health data: a proposal for a comprehensive screening tool to address health equity a systematic review. Health Aff Sch. (2024) 2. doi: 10.1093/haschl/qxae151, 39677005 PMC11642620

[144] Santini ZI Jose PE York Cornwell E Koyanagi A Nielsen L Hinrichsen C . Social disconnectedness, perceived isolation, and symptoms of depression and anxiety among older Americans (NSHAP): a longitudinal mediation analysis. Lancet Public Health. (2020) 5:e62–70. doi: 10.1016/S2468-2667(19)30230-0, 31910981

[145] Iob E Steptoe A Zaninotto P. Mental health, financial, and social outcomes among older adults with probable COVID-19 infection: a longitudinal cohort study. Proc Natl Acad Sci USA. (2022) 119:e2200816119. doi: 10.1073/pnas.2200816119, 35763577 PMC9271189

