# Supplementary material for: Persistent socio-demographic inequalities in depression among older adults in China during and after COVID-19: a systematic review and meta-analysis
Source: Front Public Health. 2026 Sep 16;14:1929179. doi: 10.3389/fpubh.2026.1929179 (PMC13625264; doi:10.3389/fpubh.2026.1929179)
Supplement: Supplementary file 1 [file Supplementary_file_1.docx]

Supplementary Material

# 1 Supplementary Tables

## 1.1 Supplementary Table 1. Preferred Reporting Items for Systematic Reviews and Meta-Analysis (PRISMA) 2020 Checklist

| **Section and Topic** | **Item #** | **Checklist item** | **Location where item is reported** |
| --- | --- | --- | --- |
| **TITLE** | | |  |
| Title | 1 | Identify the report as a systematic review. | Page 1 |
| **ABSTRACT** | | |  |
| Abstract | 2 | See the PRISMA 2020 for Abstracts checklist. | Page 1-2 |
| **INTRODUCTION** | | |  |
| Rationale | 3 | Describe the rationale for the review in the context of existing knowledge. | Page 2-3 |
| Objectives | 4 | Provide an explicit statement of the objective(s) or question(s) the review addresses. | Page 3 |
| **METHODS** | | |  |
| Eligibility criteria | 5 | Specify the inclusion and exclusion criteria for the review and how studies were grouped for the syntheses. | Page 3-4 |
| Information sources | 6 | Specify all databases, registers, websites, organizations, reference lists and other sources searched or consulted to identify studies. Specify the date when each source was last searched or consulted. | Page 3 |
| Search strategy | 7 | Present the full search strategies for all databases, registers and websites, including any filters and limits used. | Page 3  Supplementary Table 2 |
| Selection process | 8 | Specify the methods used to decide whether a study met the inclusion criteria of the review, including how many reviewers screened each record and each report retrieved, whether they worked independently, and if applicable, details of automation tools used in the process. | Page 3-4 Figure 1 |
| Data collection process | 9 | Specify the methods used to collect data from reports, including how many reviewers collected data from each report, whether they worked independently, any processes for obtaining or confirming data from study investigators, and if applicable, details of automation tools used in the process. | Page 3-4 |
| Data items | 10a | List and define all outcomes for which data were sought. Specify whether all results that were compatible with each outcome domain in each study were sought (e.g. for all measures, time points, analyses), and if not, the methods used to decide which results to collect. | Page 3-4 |
|  | 10b | List and define all other variables for which data were sought (e.g. participant and intervention characteristics, funding sources). Describe any assumptions made about any missing or unclear information. | Supplementary Table 4 |
| Study risk of bias assessment | 11 | Specify the methods used to assess risk of bias in the included studies, including details of the tool(s) used, how many reviewers assessed each study and whether they worked independently, and if applicable, details of automation tools used in the process. | Page 4-5 |
| Effect measures | 12 | Specify for each outcome the effect measure(s) (e.g. risk ratio, mean difference) used in the synthesis or presentation of results. | Page 4-5 |
| Synthesis methods | 13a | Describe the processes used to decide which studies were eligible for each synthesis (e.g. tabulating the study intervention characteristics and comparing against the planned groups for each synthesis (item #5)). | Page 3-4 |
|  | 13b | Describe any methods required to prepare the data for presentation or synthesis, such as handling of missing summary statistics, or data conversions. | Page 4-5 |
|  | 13c | Describe any methods used to tabulate or visually display results of individual studies and syntheses. | Page 4-5 |
|  | 13d | Describe any methods used to synthesize results and provide a rationale for the choice(s). If meta-analysis was performed, describe the model(s), method(s) to identify the presence and extent of statistical heterogeneity, and software package(s) used. | Page 4 |
|  | 13e | Describe any methods used to explore possible causes of heterogeneity among study results (e.g. subgroup analysis, meta-regression). | Page 4 |
|  | 13f | Describe any sensitivity analyses conducted to assess robustness of the synthesized results. | Page 4-5 |
| Reporting bias assessment | 14 | Describe any methods used to assess risk of bias due to missing results in a synthesis (arising from reporting biases). | Page 5 |
| Certainty assessment | 15 | Describe any methods used to assess certainty (or confidence) in the body of evidence for an outcome. | NA |
| **RESULTS** | | |  |
| Study selection | 16a | Describe the results of the search and selection process, from the number of records identified in the search to the number of studies included in the review, ideally using a flow diagram. | Page 5  Figure 1 |
|  | 16b | Cite studies that might appear to meet the inclusion criteria, but which were excluded, and explain why they were excluded. | Page 3-4 Figure 1 |
| Study characteristics | 17 | Cite each included study and present its characteristics. | Page 5-6,  Supplementary Table 4 |
| Risk of bias in studies | 18 | Present assessments of risk of bias for each included study. | Page 6 Supplementary Tables 3-4 |
| Results of individual studies | 19 | For all outcomes, present, for each study: (a) summary statistics for each group (where appropriate) and (b) an effect estimate and its precision (e.g. confidence/credible interval), ideally using structured Supplementary Tables or plots. | Figure 2 Supplementary Table 4 |
| Results of syntheses | 20a | For each synthesis, briefly summarize the characteristics and risk of bias among contributing studies. | Page 5-6  Supplementary Tables 3–4 |
|  | 20b | Present results of all statistical syntheses conducted. If meta-analysis was done, present for each the summary estimate and its precision (e.g. confidence/credible interval) and measures of statistical heterogeneity. If comparing groups, describe the direction of the effect. | Page 5-7  Figures 3-5 Supplementary Tables 5 and 7-8  Supplementary Figures 1-5 |
|  | 20c | Present results of all investigations of possible causes of heterogeneity among study results. | Page 5-6  Supplementary Tables 5-6 |
|  | 20d | Present results of all sensitivity analyses conducted to assess the robustness of the synthesized results. | Page 6  Supplementary Tables 9-10 |
| Reporting biases | 21 | Present assessments of risk of bias due to missing results (arising from reporting biases) for each synthesis assessed. | Page 6 |
| Certainty of evidence | 22 | Present assessments of certainty (or confidence) in the body of evidence for each outcome assessed. | NA |
| **DISCUSSION** | | |  |
| Discussion | 23a | Provide a general interpretation of the results in the context of other evidence. | Page 7-8 |
|  | 23b | Discuss any limitations of the evidence included in the review. | Page 7-8 |
|  | 23c | Discuss any limitations of the review processes used. | Page 9 |
|  | 23d | Discuss implications of the results for practice, policy, and future research. | Page 9 |
| **OTHER INFORMATION** | | |  |
| Registration and protocol | 24a | Provide registration information for the review, including register name and registration number, or state that the review was not registered. | Page 3 |
|  | 24b | Indicate where the review protocol can be accessed, or state that a protocol was not prepared. | Page 2 |
|  | 24c | Describe and explain any amendments to information provided at registration or in the protocol. | Page 3 |
| Support | 25 | Describe sources of financial or non-financial support for the review, and the role of the funders or sponsors in the review. | Page 10 |
| Competing interests | 26 | Declare any competing interests of review authors. | Page 10 |
| Availability of data, code and other materials | 27 | Report which of the following are publicly available and where they can be found: template data collection forms; data extracted from included studies; data used for all analyses; analytic code; any other materials used in the review. | Page 9-10 |

## 1.2 Supplementary Table 2. Search strategy to identify studies reporting the prevalence of depression in older adults

| **Database** | **Access date** | **Search terms** |
| --- | --- | --- |
| **CNKI** | 1^st^Jan, 2020  -15^th^ June, 2026 | (KY=('老年人'+'老年'+'老年人群'+'老人')) AND (KY=('抑郁'+'抑郁症'+'抑郁症状')) |
| **CQVIP** | 1^st^Jan, 2020  -15^th^ June, 2026 | (K=(老年人 OR 老年 OR 老年人群 OR 老人)) AND (K=(抑郁 OR 抑郁症 OR 抑郁症状)) |
| **Wanfang** | 1^st^Jan, 2020  -15^th^ June, 2026 | (关键词:(“老年人” or “老年” or “老年人群” or “老人”) and 关键词:(“抑郁” or “抑郁症” or “抑郁症状”)) |
| **CBMdisc** | 1^st^Jan, 2020  -15^th^ June, 2026 | ("老年人"[关键词:智能] OR "老年"[关键词:智能] OR "老年人群"[关键词:智能] OR "老人"[关键词:智能]) AND ("抑郁"[关键词:智能] OR "抑郁症"[关键词:智能] OR "抑郁症状"[关键词:智能]) |
| **PubMed** | 1^st^Jan, 2020  -15^th^ June, 2026 | (elder OR elderly OR "old population" OR aged OR "old adults" OR "older adults" OR geriatrics OR "late life" ) AND (depression OR depressive OR "depressive disorder" OR "depressive symptom" OR depressed OR "depressed mental" OR distress OR "mental distress" OR dysthymia OR "dysthymic disorder" OR "late-life depression") AND (China OR Chinese) |
| **Embase** | 1^st^Jan, 2020  -15^th^ June, 2026 | (elder:kw OR elderly:kw OR 'old population':kw OR aged:kw OR 'old adults':kw OR 'older adults':kw OR geriatrics:kw OR 'late life':kw) AND (depression:kw OR depressive:kw OR 'depressive disorder':kw OR 'depressive symptom':kw OR depressed:kw OR 'depressed mental':kw OR distress:kw OR 'mental distress':kw OR dysthymia:kw OR 'dysthymic disorder':kw OR 'late-life depression':kw) AND (China:kw OR Chinese:kw) |
| **Web of Science** | 1^st^Jan, 2020  -15^th^ June, 2026 | (KP=(elder OR elderly OR "old population" OR aged OR "old adults" OR "older adults" OR geriatrics OR "late life")) AND (KP=(depression OR depressive OR "depressive disorder" OR "depressive symptom" OR depressed OR "depressed mental" OR distress OR "mental distress" OR dysthymia OR "dysthymic disorder" OR "late-life depression")) AND (KP=(China OR Chinese)) |
| **Scopus** | 1^st^Jan, 2020  -15^th^ June, 2026 | KEY(elder OR elderly OR "old population" OR aged OR "old adults" OR "older adults" OR geriatrics OR "late life") AND KEY(depression OR depressive OR "depressive disorder" OR "depressive symptom" OR depressed OR "depressed mental" OR distress OR "mental distress" OR dysthymia OR "dysthymic disorder" OR "late-life depression") AND KEY(China OR Chinese) |
| **PsycINFO** | 1^st^Jan, 2020  -15^th^ June, 2026 | KW ( elder OR elderly OR "old population" OR aged OR "old adults" OR "older adults" OR geriatrics OR "late life" ) AND KW ( depression OR depressive OR "depressive disorder" OR "depressive symptom" OR depressed OR "depressed mental" OR distress OR "mental distress" OR dysthymia OR "dysthymic disorder" OR "late-life depression" ) AND KW ( China OR Chinese ) |

## 1.3 Supplementary Table 3. Quality scores for assessing the risk of bias in the included articles (n = 79)*

| Number | Study ID | Q1 | Q2 | Q3 | Q4 | Q5 | Q6 | Q7 | Q8 | Q9 | Q10 | Q11 | Quality Score |
| --- | --- | --- | --- | --- | --- | --- | --- | --- | --- | --- | --- | --- | --- |
| 1 | Chen BY(2023) | YES | YES | YES | YES | NO | YES | NO | YES | NO | YES | NO | 7 |
| 2 | Qin Z (2022) | YES | YES | YES | YES | NO | YES | YES | YES | NO | YES | NO | 8 |
| 3 | Yu J (2022) | YES | YES | YES | YES | NO | YES | YES | YES | NO | YES | NO | 8 |
| 4 | Qin Y (2022) | YES | YES | YES | YES | NO | YES | YES | YES | NO | YES | NO | 8 |
| 5 | Liu J (2022) | YES | YES | YES | YES | NO | YES | YES | YES | NO | YES | NO | 8 |
| 6 | Cui C(2022) | YES | YES | YES | YES | NO | YES | YES | NO | NO | YES | NO | 7 |
| 7 | Xu K (2022) | YES | YES | YES | YES | NO | YES | YES | NO | YES | YES | NO | 8 |
| 8 | Liu C (2022) | YES | YES | YES | YES | NO | YES | YES | YES | NO | YES | NO | 8 |
| 9 | Lu C (2022) | YES | YES | YES | YES | NO | YES | YES | YES | NO | YES | NO | 8 |
| 10 | Lu L (2023) | YES | YES | YES | YES | NO | YES | YES | YES | NO | YES | NO | 8 |
| 11 | Hao R (2023) | YES | YES | YES | YES | NO | YES | YES | YES | NO | YES | NO | 8 |
| 12 | Li Y (2022) | YES | YES | YES | YES | NO | YES | NO | YES | NO | YES | NO | 7 |
| 13 | Li R (2022) | YES | YES | YES | YES | NO | YES | YES | YES | NO | YES | NO | 8 |
| 14 | Li S (2022) | YES | YES | YES | YES | NO | YES | NO | NO | NO | YES | NO | 6 |
| 15 | Ding K (2022) | YES | YES | YES | YES | NO | YES | NO | YES | NO | YES | NO | 7 |
| 16 | Hou P (2022) | YES | YES | YES | YES | NO | YES | YES | YES | NO | YES | NO | 8 |
| 17 | Mao S (2022) | YES | YES | YES | YES | NO | YES | NO | YES | NO | YES | NO | 7 |
| 18 | Liu W (2022) | YES | YES | YES | YES | NO | YES | NO | YES | NO | YES | NO | 7 |
| 19 | Wang S (2021) | YES | YES | YES | YES | NO | YES | NO | YES | NO | YES | NO | 7 |
| 20 | Liu A (2021) | YES | YES | YES | YES | NO | YES | YES | YES | NO | YES | NO | 8 |
| 21 | Zhou R (2021) | YES | YES | YES | YES | NO | YES | YES | YES | YES | YES | NO | 9 |
| 22 | Liang W (2021) | YES | YES | YES | YES | NO | YES | YES | YES | NO | YES | NO | 8 |
| 23 | Wang G (2020) | YES | YES | YES | YES | NO | YES | NO | YES | NO | YES | NO | 7 |
| 24 | Wang S (2020) | YES | YES | YES | YES | NO | YES | NO | YES | NO | YES | NO | 7 |
| 25 | He L (2022) | YES | YES | YES | YES | NO | YES | YES | YES | NO | YES | NO | 8 |
| 26 | Li H (2022) | YES | YES | YES | YES | NO | YES | YES | YES | YES | YES | NO | 9 |
| 27 | Yu H (2024) | YES | YES | YES | YES | NO | NO | YES | YES | YES | YES | NO | 8 |
| 28 | Zhao Z (2024) | YES | YES | YES | YES | NO | YES | YES | YES | YES | YES | NO | 9 |
| 29 | Liu M (2024) | YES | YES | YES | YES | NO | YES | YES | YES | NO | YES | NO | 8 |
| 30 | Zong L (2022) | YES | YES | YES | YES | NO | YES | YES | YES | YES | YES | NO | 9 |
| 31 | Zhang Y (2024) | YES | YES | YES | YES | NO | YES | NO | YES | NO | YES | NO | 7 |
| 32 | Liu Y (2023) | YES | YES | YES | YES | NO | YES | YES | YES | YES | YES | NO | 9 |
| 33 | Yang L (2024) | YES | YES | YES | NO | NO | NO | YES | YES | YES | YES | NO | 7 |
| 34 | Liu S (2024) | YES | YES | YES | YES | NO | NO | YES | YES | NO | YES | NO | 7 |
| 35 | Huang W (2024) | YES | YES | YES | YES | NO | YES | YES | YES | YES | YES | NO | 9 |
| 36 | Zhao Q (2023) | YES | YES | YES | YES | NO | NO | YES | NO | NO | YES | NO | 6 |
| 37 | Li Q (2024) | YES | YES | YES | NO | NO | NO | YES | YES | YES | YES | NO | 7 |
| 38 | Su H (2023) | YES | YES | YES | YES | NO | YES | NO | YES | NO | YES | NO | 7 |
| 39 | Lin Y (2023) | YES | YES | YES | YES | NO | YES | NO | YES | NO | YES | NO | 7 |
| 40 | Yang J (2023) | YES | YES | YES | YES | NO | YES | NO | YES | NO | YES | NO | 7 |
| 41 | Ge H (2023) | YES | YES | NO | YES | NO | NO | YES | YES | YES | YES | NO | 7 |
| 42 | Jiang Y (2024) | YES | YES | YES | YES | NO | YES | YES | YES | YES | YES | NO | 9 |
| 43 | Zhang Y (2024) | YES | YES | YES | YES | NO | YES | NO | YES | NO | YES | NO | 7 |
| 44 | Hu Q (2023) | YES | YES | YES | YES | NO | YES | NO | NO | NO | YES | NO | 6 |
| 45 | Gan S (2024) | YES | YES | YES | YES | NO | YES | YES | NO | YES | YES | NO | 8 |
| 46 | Xiong H (2024) | YES | YES | YES | YES | NO | YES | NO | YES | NO | YES | NO | 7 |
| 47 | Peng X (2024) | YES | YES | YES | YES | NO | YES | YES | NO | YES | YES | NO | 8 |
| 48 | He Z (2024) | YES | YES | YES | YES | NO | YES | YES | YES | YES | YES | NO | 9 |
| 49 | Zeng H (2025) | YES | YES | YES | YES | NO | YES | YES | YES | YES | YES | NO | 9 |
| 50 | Wang X (2025) | YES | YES | YES | YES | NO | YES | YES | YES | YES | YES | NO | 9 |
| 51 | Chen Y (2025) | YES | YES | YES | YES | NO | YES | YES | YES | NO | YES | NO | 8 |
| 52 | Wu J (2025) | YES | YES | YES | YES | NO | YES | YES | NO | NO | YES | NO | 7 |
| 53 | Zhang Y (2025) | YES | YES | YES | YES | NO | YES | YES | YES | NO | YES | NO | 8 |
| 54 | Yang T (2025) | YES | YES | YES | YES | NO | YES | YES | YES | YES | YES | YES | 10 |
| 55 | Zhou L (2025) | YES | YES | YES | YES | NO | YES | YES | YES | NO | YES | NO | 8 |
| 56 | Du S (2025) | YES | YES | YES | YES | NO | YES | YES | YES | NO | YES | NO | 8 |
| 57 | Liu D (2025) | YES | YES | YES | YES | NO | YES | NO | YES | NO | YES | NO | 7 |
| 58 | Wei T (2025) | YES | YES | YES | YES | NO | YES | YES | YES | NO | YES | NO | 8 |
| 59 | Wei S (2025) | YES | YES | YES | YES | NO | YES | YES | YES | NO | YES | NO | 8 |
| 60 | Liu S (2025) | YES | YES | YES | YES | NO | YES | YES | YES | NO | YES | NO | 8 |
| 61 | Liu C (2025) | YES | YES | YES | YES | NO | YES | YES | YES | NO | YES | NO | 8 |
| 62 | Li D (2025) | YES | YES | YES | YES | NO | YES | YES | YES | NO | YES | NO | 8 |
| 63 | Zhao H (2025) | YES | YES | YES | YES | NO | YES | NO | YES | NO | YES | NO | 7 |
| 64 | Feng W (2025) | YES | YES | YES | YES | NO | YES | NO | YES | NO | YES | NO | 7 |
| 65 | Lu J (2025) | YES | YES | YES | YES | NO | YES | YES | YES | NO | YES | NO | 8 |
| 66 | Chen R (2025) | YES | YES | YES | YES | NO | YES | YES | YES | NO | YES | NO | 8 |
| 67 | Dong P (2025) | YES | YES | YES | YES | NO | YES | YES | YES | YES | YES | NO | 9 |
| 68 | Dong P (2025) | YES | YES | YES | YES | NO | YES | NO | YES | YES | YES | NO | 8 |
| 69 | Wu S (2025) | YES | YES | YES | YES | NO | YES | YES | YES | YES | YES | NO | 9 |
| 70 | Zhang X (2025) | YES | YES | YES | YES | NO | YES | YES | YES | NO | YES | NO | 8 |
| 71 | Lan Y (2025) | YES | YES | YES | YES | NO | YES | YES | YES | NO | YES | NO | 8 |
| 72 | Wang Y (2025) | YES | YES | YES | YES | NO | YES | YES | YES | NO | YES | NO | 8 |
| 73 | Jing Y (2025) | YES | YES | YES | YES | NO | YES | YES | YES | NO | YES | NO | 8 |
| 74 | Huang Y (2025) | YES | YES | YES | YES | NO | YES | YES | YES | NO | YES | NO | 8 |
| 75 | Tian Y (2025) | YES | YES | YES | YES | NO | YES | YES | YES | YES | YES | NO | 9 |
| 76 | Lin X (2025) | YES | YES | YES | YES | NO | YES | YES | YES | YES | YES | NO | 9 |
| 77 | Dong M (2026) | YES | YES | YES | YES | NO | YES | NO | YES | NO | YES | NO | 7 |
| 78 | Zhang J (2026) | YES | YES | YES | YES | NO | YES | YES | YES | NO | YES | NO | 8 |
| 79 | Xu N (2026) | YES | YES | YES | YES | NO | YES | YES | YES | NO | YES | NO | 8 |

## 1.4 Supplementary Table 4. Characteristics of the 79 studies included in the current study

| Number | Study ID | Publication Language | Region | Investigation year | Sample(N) | Case (N, %) | Male (N, %) | Urban (N, %) | Married(N,%) | Live with others (N, %) | Senior secondary school and above  (N, %) |
| --- | --- | --- | --- | --- | --- | --- | --- | --- | --- | --- | --- |
| 1 | Chen B (2023) | CH | Western | 2022 | 1418 | 119(8.39) | 669(47.18) | NA | 1089(76.80) | 1315(92.74) | NA |
| 2 | Qin Z (2022) | CH | Eastern | 2021 | 1946 | 316(16.24) | 979(50.31) | 1254(64.44) | 1599(82.17) | 1844(94.76) | NA |
| 3 | Yu J (2022) | CH | Western | 2021 | 1937 | 760(39.24) | 919(47.44) | 892(46.05) | 1507(77.80) | 1644(84.87) | 183(36.07) |
| 4 | Qin Y (2022) | CH | Eastern | 2020 | 1004 | 93(9.26) | 443(44.12) | NA | 789(78.59) | 918(91.43) | NA |
| 5 | Liu J (2022) | CH | Central | 2020 | 267 | 113(42.32) | 124(46.44) | NA | 243(91.01) | 240(89.89) | 9(11.11) |
| 6 | Cui (2022) | CH | Western | 2021 | 315 | 55(17.46) | NA | NA | NA | NA | NA |
| 7 | Xu K (2022) | CH | Central | 2021 | 235 | 65(27.66) | 78(33.19) | NA | NA | NA | NA |
| 8 | Liu C (2022) | CH | Eastern | 2021 | 411 | 165(40.15) | 193(46.96) | NA | NA | NA | NA |
| 9 | Lu C (2022) | CH | Eastern | 2020 | 2102 | 779(37.06) | 1078(51.28) | NA | 1616(76.88) | NA | 898(33.30) |
| 10 | Lu L (2023) | EN | Central | 2021 | 1173 | 438(37.34) | 544(46.38) | NA | NA | NA | NA |
| 11 | Hao R (2023) | EN | Mixed | 2021 | 1122 | 529(47.15) | 569(50.71) | NA | NA | NA | NA |
| 12 | Li Y (2022) | EN | Mixed | 2021 | 1147 | 551(48.04) | 581(50.65) | NA | NA | NA | NA |
| 13 | Li R (2022) | EN | Eastern | 2020 | 288 | 37(12.85) | 136(47.22) | NA | NA | NA | NA |
| 14 | Li S (2022) | EN | Eastern | 2021 | 383 | 39(10.18) | NA | NA | NA | NA | NA |
| 15 | Ding K (2022) | EN | Eastern | 2021 | 506 | 84(16.60) | 196(38.74) | NA | NA | NA | NA |
| 16 | Hou P (2022) | EN | Eastern | 2020 | 527 | 52(9.87) | 261(49.53) | NA | NA | NA | NA |
| 17 | Mao S (2022) | EN | Eastern | 2020 | 472 | 356(75.42) | 206(43.64) | NA | NA | NA | NA |
| 18 | Liu W (2022) | EN | Mixed | 2020 | 1753 | 90(5.13) | NA | NA | NA | NA | NA |
| 19 | Wang S (2021) | EN | Mixed | 2020 | 5701 | 853(14.96) | NA | NA | NA | NA | NA |
| 20 | Liu A (2021) | EN | Central | 2020 | 470 | 66(14.04) | 197(41.91) | NA | NA | 388(82.55) | 63(1.59) |
| 21 | Zhou R (2021) | EN | Mixed | 2020 | 1278 | 177(13.85) | 571(44.68) | 588(46.01) | 954(74.65) | 1156(90.45) | 357(7.84) |
| 22 | Liang W (2021) | EN | Central | 2020 | 516 | 159(30.81) | 217(42.05) | NA | NA | NA | NA |
| 23 | Wang G (2020) | EN | Mixed | 2020 | 852 | 65(7.63) | NA | NA | NA | NA | NA |
| 24 | Wang S (2020) | EN | Mixed | 2020 | 3560 | 378(10.62) | NA | NA | NA | NA | NA |
| 25 | He L (2022) | EN | Western | 2022 | 2026 | 609(30.06) | NA | NA | NA | NA | NA |
| 26 | Li H (2022) | EN | Eastern | 2021 | 613 | 42(6.85) | 165(26.92) | 88(14.36) | 539(87.93) | NA | 109(9.17) |
| 27 | Yu H (2024) | CH | Eastern | 2020 | 3767 | 50(7.67) | 1502(39.87) | NA | NA | NA | NA |
| 28 | Zhao Z (2024) | CH | Eastern | 2022 | 3575 | 573(16.92) | 1594(44.59) | NA | NA | NA | NA |
| 29 | Liu M (2024) | CH | Western | 2022 | 606 | 85(14.03) | 274(45.21) | NA | 430(70.96) | 537(88.61) | NA |
| 30 | Zong L (2022) | CH | Eastern | 2021 | 3192 | 198(6.20) | 1211(37.94) | NA | 2505(78.48) | NA | 332(3.92) |
| 31 | Zhang Y (2024) | CH | Mixed | 2020 | 8240 | 3373(40.93) | 4167(50.57) | NA | 6568(79.71) | NA | 911(23.16) |
| 32 | Liu Y (2023) | CH | Western | 2022 | 395 | 30(7.60) | 141(35.70) | NA | NA | NA | NA |
| 33 | Yang L (2024) | CH | Central | 2023 | 1851 | 915(49.40) | 946(51.11) | NA | 1346(72.72) | NA | NA |
| 34 | Liu S (2024) | CH | Western | 2022 | 527 | 122(23.15) | 280(53.13) | NA | 463(87.86) | NA | NA |
| 35 | Huang W (2024) | CH | Central | 2023 | 6004 | 2048(34.11) | 2914(48.53) | 2848(47.44) | 3500(58.29) | NA | NA |
| 36 | Zhao Q (2023) | CH | Eastern | 2021 | 537 | 185(34.50) | 288(53.63) | NA | NA | NA | NA |
| 37 | Li Q (2024) | CH | Western | 2021 | 1409 | 733(52.00) | 690(48.97) | NA | NA | NA | NA |
| 38 | Su H (2023) | CH | Central | 2023 | 388 | 70(18.04) | NA | NA | NA | NA | NA |
| 39 | Lin Y (2023) | CH | Central | 2022 | 665 | 72(10.80) | 281(42.26) | NA | 503(75.64) | NA | NA |
| 40 | Yang J (2023) | CH | Eastern | 2020 | 2112 | 626(29.60) | 1032(48.86) | NA | 1623(76.85) | NA | 902(33.37) |
| 41 | Ge H (2023) | CH | Mixed | 2020 | 4361 | 1282(29.40) | 2286(52.42) | 2191(50.24) | NA | NA | NA |
| 42 | Jiang Y (2024) | EN | Mixed | 2022 | 648 | 130(20.10) | NA | NA | NA | NA | NA |
| 43 | Zhang Y (2024) | EN | Central | 2022 | 1866 | 255(13.67) | 619(33.17) | NA | NA | NA | NA |
| 44 | Hu Q (2023) | EN | Eastern | 2021 | 471 | 65(13.80) | 187(39.70) | NA | NA | NA | NA |
| 45 | Gan S (2024) | EN | Mixed | 2020 | 317 | 50(15.77) | 74(23.34) | NA | NA | NA | NA |
| 46 | Xiong H (2024) | EN | Mixed | 2021 | 165 | 34(20.61) | NA | NA | NA | NA | NA |
| 47 | Peng X (2024) | EN | Eastern | 2020 | 5331 | 554(10.39) | 2472(46.37) | NA | NA | NA | NA |
| 48 | He Z (2024) | EN | Eastern | 2022 | 8436 | 212(2.51) | NA | NA | NA | NA | NA |
| 49 | Zeng H (2025) | EN | Central | 2023 | 8388 | 1376(16.40) | 4483(53.45) | NA | 6467(77.10) | 7128(84.98) | 854(10.66) |
| 50 | Wang X (2025) | EN | Mixed | 2022 | 2584 | 1026(39.71) | NA | NA | NA | NA | NA |
| 51 | Chen Y (2025) | CH | Central | 2023 | 950 | 478(50.32) | NA | NA | NA | NA | NA |
| 52 | Wu J (2025) | CH | Eastern | 2023 | 5069 | 656(12.94) | NA | NA | NA | NA | NA |
| 53 | Zhang Y (2025) | CH | Eastern | 2022 | 3052 | 629(20.61) | NA | NA | NA | NA | NA |
| 54 | Yang T (2025) | EN | Mixed | 2020 | 2248 | 789(35.10) | NA | NA | NA | NA | NA |
| 55 | Zhou L (2025) | EN | Western | 2023 | 538 | 247(45.91) | NA | NA | NA | NA | NA |
| 56 | Du S (2025) | EN | Western | 2022-2023 | 1377 | 421(30.57) | NA | NA | NA | NA | NA |
| 57 | Liu D (2025) | EN | Western | 2023 | 2259 | 167(7.39) | NA | NA | NA | NA | NA |
| 58 | TianW (2025) | EN | Mixed | 2023 | 2516 | 172(6.84) | NA | NA | NA | NA | NA |
| 59 | Wei S (2025) | CH | Western | 2023 | 1051 | 213(20.27) | NA | NA | NA | NA | NA |
| 60 | Liu S (2025) | CH | Mixed | 2021 | 4246 | 238(5.61) | 2034(47.90) | 1592(37.49) | 2405(56.64) | 3338(78.62) | NA |
| 61 | Liu C (2025) | CH | Eastern | 2022 | 1133 | 304(26.83) | NA | NA | NA | NA | NA |
| 62 | Li D (2025) | CH | Western | 2023 | 1483 | 403(27.17) | NA | NA | NA | NA | NA |
| 63 | Zhao H (2025) | CH | Western | 2023 | 893 | 208(23.29) | NA | NA | NA | NA | NA |
| 64 | Feng W (2025) | CH | Western | 2023 | 13286 | 1954(14.71) | 5814(43.76) | NA | 10527(79.23) | 10740(80.84) | 3071(9.96) |
| 65 | Lu J (2025) | CH | Western | 2022-2023 | 983 | 190(19.33) | 521(53.00) | 771(78.43) | 664(67.55) | 686(69.79) | NA |
| 66 | Chen R (2025) | CH | Eastern | 2023 | 2111 | 478(22.64) | 1079(51.11) | 1081(51.21) | 1842(87.26) | 1782(84.41) | 428(10.75) |
| 67 | Dong P (2025) | EN | Eastern | 2022 | 10369 | 2607(25.14) | 4590(44.27) | 5337(51.47) | 7195(69.39) | 9615(92.73) | NA |
| 68 | Dong P (2025) | EN | Mixed | 2024 | 872 | 242(27.75) | NA | NA | NA | NA | NA |
| 69 | Wu S (2025) | EN | Eastern | 2024 | 1550 | 247(15.94) | 660(42.58) | 521(33.61) | 1049(67.67) | 1362(87.87) | NA |
| 70 | Zhang X (2025) | CH | Eastern | 2022 | 23956 | 1317(5.50) | 11460(47.84) | 19754(82.46) | 20334(84.88) | 21875(91.31) | NA |
| 71 | Lan Y (2025) | CH | Western | 2025 | 466 | 88(18.88) | NA | NA | NA | NA | NA |
| 72 | Wang Y (2025) | CH | Western | 2023 | 490 | 187(38.16) | 245(50.00) | NA | NA | NA | NA |
| 73 | Jing Y (2025) | CH | Eastern | 2021 | 2329 | 184(7.90) | NA | NA | NA | NA | NA |
| 74 | Huang Y (2025) | CH | Mixed | 2023 | 1035 | 105(10.14) | NA | NA | NA | NA | NA |
| 75 | Tian Y (2025) | CH | Eastern | 2022 | 328 | 49(14.94) | 142(43.29) | NA | 248(75.61) | 245(74.70) | NA |
| 76 | Lin X (2025) | CH | Central | 2024 | 6305 | 1939(30.75) | 3013(47.79) | NA | 4939(78.33) | 4200(66.61) | 126(2.00) |
| 77 | Dong M (2026) | CH | Eastern | 2021 | 4001 | 403(10.07) | NA | NA | NA | NA | NA |
| 78 | Zhang J (2026) | CH | Mixed | 2022 | 3666 | 923(25.18) | NA | NA | NA | NA | NA |
| 79 | Xu N (2026) | EN | Western | 2022 | 2697 | 618(22.91) | NA | NA | NA | NA | NA |

Abbreviations: NA = Not Applicable.

**79 Included studies**

# 1. Chen BY, Mo Y, Chen LJ, Zhang ZJ, Wang FL, Cai L. [Prevalence of depression and its relationship with social support among the Bai ethnic minority elderly in rural Yunnan Province]. Chinese Rural Health Service Administration. (2023) 43(2):141-146. doi:10.19955/j.cnki.1005-5916.2023.02.012 [in Chinese].

# 2. Qin ZL, Deng SS, Li C. [Analysis of depression status and its influence on the quality of life of the elderly in western Guangxi]. Journal of Youjiang Medical University for Nationalities. (2022) 44(6):881-886. [in Chinese].

# 3. Yu JQ, Wu YJ, Zhang Y, He YZ, Cai YT, Zheng JZ, et al. [Correlation between depressive symptoms and health promotion behavior in the elderly: based on a structural equation model]. Chinese Journal of Disease Control & Prevention. (2022) 26(9):1072-1077. [in Chinese]. doi:10.16462/j.cnki.zhjbkz.2022.09.014.

# 4. Qin YW, Li HJ, Ying X, Tian FY, Li SB, Wan LP, et al. [Analysis on status and influencing factors of depressive symptoms among the elderly aged 65 years and above in rural areas, Linyi city]. Preventive Medicine Tribune. (2022) 28(3):161-165. [in Chinese]. doi:10.16406/j.pmt.issn.1672-9153.2022.03.013.

# 5. Liu JX, Luo JS, Ji SQ, Wang C, Zeng ML, Li YL, et al. [Depression and its influencing factors in rural poor elderly in western Hunan province]. Journal of Community Medicine. (2022) 20(22):1243-1248. [in Chinese]. doi:10.19790/j.cnki.JCM.2022.22.01.

# 6. Cui CH, Dong A, Yang Q. [Mediating effect of leisure activities on cognitive frailty and depression in community-dwelling elderly]. Chinese Journal of Multiple Organ Diseases in the Elderly. (2022) 21(1):45-48. [in Chinese].

# 7. Xu K. [Impact of COVID-19 cognition on depression among older adults during the normalization of epidemic prevention and control: the chain mediating effect of discrimination perception and risk perception]. China Journal of Health Psychology. (2022) 30(10):1496-1501. [in Chinese]. doi:10.13342/j.cnki.cjhp.2022.10.011.

# 8. Liu CC, Chen LQ, Xie BQ, Chen Y, Wang JL, Xu HQ. [Prevalence and determinants of social isolation among community-living oldest-old adults]. Journal of Nursing Science. (2022) 37(13):98-102. [in Chinese].

# 9. Lu C, Guan T, Yang J, Zhang DM, Lin XH. [Residence patterns, level of community service and depressive symptoms of older adults in Shenzhen]. Scientific Research on Aging. (2022) 10(5):44-57. [in Chinese].

# 10. Lu L, Shen H, Tan L, Huang Q, Chen Q, Liang M, et al. Prevalence and factors associated with anxiety and depression among community-dwelling older adults in Hunan, China: a cross-sectional study. BMC Psychiatry. (2023) 23(1):107. doi:10.1186/s12888-023-04583-5

# 11. Hao R, Jin H, Zuo J, Wu Y, Sun X, Hu J. The multiple mediating effect of family health and perceived social support on depressive symptoms in older adults: A cross-sectional national survey in China. J Affect Disord. (2023) 327:348-354. doi:10.1016/j.jad.2023.01.097

# 12. Li Y, Lin Z, Wu Y. Exploring depression among the elderly during the COVID-19 pandemic: the effects of the big five, media use, and perceived social support. Int J Environ Res Public Health. (2022) 19(20):13534. doi:10.3390/ijerph192013534

# 13. Li R, Zong ZY, Gu XX, Wang DN, Dong C, Sun C, et al. Higher dietary diversity as a protective factor against depression among older adults in China: a cross-sectional study. Ann Palliat Med. (2022) 11(4):1278289. doi:10.21037/apm-21-1449

# 14. Li S, Guo B, Lu X, Yang Q, Zhu H, Ji Y, et al. Investigation of mental health literacy and status of residents during the re-outbreak of COVID-19 in China. Front Public Health. (2022) 10:895553. doi:10.3389/fpubh.2022.895553

# 15. Ding KR, Wang SB, Xu WQ, Lin LH, Liao DD, Chen HB, et al. Low mental health literacy and its association with depression, anxiety and poor sleep quality in Chinese elderly. Asia Pac Psychiatry. (2022) 14(4):e12520. doi:10.1111/appy.12520

# 16. Hou P, Xue H, Zhang Y, Ping Y, Zheng Y, Wang Y, et al. Mediating effect of loneliness in the relationship between depressive symptoms and cognitive frailty in community-dwelling older adults. Brain Sci. (2022) 12(10):1341. doi:10.3390/brainsci12101341

# 17. Mao S, Lu N, Xiao C. Perceived neighborhood environment and depressive symptoms among older adults living in urban China: the mediator role of social capital. Health Soc Care Community. (2022)30(5):e1977-e1990. doi:10.1111/hsc.13631

# 18. Liu W, Yu F, Geldsetzer P, Yang J, Wang Z, Golden T, et al. Prevalence of depression in China during the early stage of the COVID-19 pandemic: a cross-sectional study in an online survey sample. BMJ Open. (2022) 12(3):e056667. doi:10.1136/bmjopen-2021-056667

# 19. Wang S, Zhang Y, Guan Y, Ding W, Meng Y, Hu H, et al. A nationwide evaluation of the prevalence of and risk factors associated with anxiety, depression and insomnia symptoms during the return-to-work period of coronavirus disease 2019 in China. Soc Psychiatry Psychiatr Epidemiol. (2021) 56(7):1-12. doi:10.1007/s00127-021-02046-4

# 20. Liu A, Peng Y, Zhu W, Zhang Y, Ge S, Zhou Y, et al. Analysis of factors associated with depression in community-dwelling older adults in Wuhan, China. Front Aging Neurosci. (2021) 13:743193. doi:10.3389/fnagi.2021.743193

# 21. Zhou R, Chen H, Zhu L, Chen Y, Chen B, Li Y, et al. Mental health status of the elderly Chinese population during COVID-19: an online cross-sectional study. Front Psychiatry. (2021) 12:645938. doi:10.3389/fpsyt.2021.645938

# 22. Liang W, Duan Y, Shang B, Hu C, Baker JS, Lin Z, et al. Precautionary behavior and depression in older adults during the COVID-19 pandemic: an online cross-sectional study in Hubei, China. Int J Environ Res Public Health. (2021) 18(4):1853. doi:10.3390/ijerph18041853

# 23. Wang GY, Tang SF. Perceived psychosocial health and its socio-demographic correlates in times of the COVID-19 pandemic: a community-based online study in China. Infect Dis Poverty. (2020) 9(1):59-68. doi:10.1186/s40249-020-00770-8

# 24. Wang S, Zhang Y, Ding W, Meng Y, Hu H, Liu Z, et al. Psychological distress and sleep problems when people are under interpersonal isolation during an epidemic: a nationwide multicenter cross-sectional study. Eur Psychiatry. (2020) 63(1):e77. doi:10.1192/j.eurpsy.2020.78

# 25. He L, Wang J, Wang F, Zhang L, Liu Y, Xu F. Depression symptoms and quality of life in empty-nest elderly among Chengdu: A cross-sectional study. Front Psychiatry. (2022) 13:1003261. doi:10.3389/fpsyt.2022.1003261

# 26. Li H, Pang M, Wang J, Xu J, Kong F. Effects of health service utilization and informal social support on depression, anxiety, and stress among the internal migrant elderly following children in Weifang, China. Int J Environ Res Public Health. (2022) 19(22):14640. doi:10.3390/ijerph192214640

# 27. Yu HD, Pan MM, Gu HQ, Liu T, Dai J, Liu LH, et al. [Correlation analysis between depressive symptoms, cognitive status and nutritional metabolism among community-dwelling older adults]. Chinese General Practice. (2024) 27(28):3540-3545. [in Chinese].

# 28. Zhao ZH, Wang LY, Su S, Li WX. [Correlation analysis between cognitive function and depression and anxiety symptoms in community-dwelling older adults]. Guoji Jingshenbingxue Zazhi. (2024) ;51(4):1154-1160. [in Chinese]. doi:10.13479/j.cnki.jip.2024.04.005.

# 29. Liu M, Wang XY, Duan Y, Gao XF. [Study on the prevalence and influencing factors of depression among older adults in rural areas of Sichuan Province]. Modern Preventive Medicine. (2024) 51(16). doi:10.20043/j.cnki.MPM.202310259. [in Chinese]. doi:10.20043/j.cnki.MPM.202310259.

# 30. Zong LX, Ge MH. [Establishment and validation of a multifactor risk prediction model for depression among elderly residents in Weifang]. Journal of Psychiatry. (2022) 35(5):529-534. [in Chinese].

# 31. Zhang Y, Jing Q, Guo TT, Zhao XQ, Dong ZW, Ji LH, et al. [Analysis of the mediating effect of daily living ability on the relationship between sleep duration and depressive symptoms in the elderly]. Modern Preventive Medicine. (2024) 51(17):3146-3150,3156. [in Chinese]. doi:10.20043/j.cnki.MPM.202405449.

# 32. Liu YT, Chen FH, Yan P, Zhang XH, Li H, Yang Y, et al. [Mediating effect of social support on anxiety, depression and quality of life in older adults]. International Journal of Geriatrics. (2023) 44(3):330-333. [in Chinese].

# 33. Yang L, Guo D, Wang J, Guo YT, Li ZY. [Prevalence and determinants of depression among older adults relocated for poverty alleviation in Shanxi Province: a cross-sectional survey]. Chinese Journal of Public Health. (2024) 40(6):686-690. [in Chinese].

# 34. Liu SB, Li XY, Sun GH. [The status of anxiety and depression and their influencing factors among older adults in a county community]. Guoji Jingshenbingxue Zazhi. (2024) 51(3):788-791. [in Chinese]. doi:10.13479/j.cnki.jip.2024.03.039.

# 35. Huang W, Qiu ZJ. [Survey on the current status of depression among elderly residents in Hunan Province and construction of a risk prediction model]. Journal of Yueyang Vocational and Technical College. (2024) 39(1):71-78. [in Chinese]. doi:10.13947/j.cnki.yyzyxb.2024.01.012.

# 36. Zhao QP, Cui Y, Zhang XY, Zheng M, Liu SS, Wang FL. [Relationship of resilience and family intergenerational solidarity with frailty and depression among middle-aged and older adults]. Journal of Nurses Training. (2023) 38(14):1272-1275. [in Chinese]. doi:10.16821/j.cnki.hsjx.2023.14.005.

# 37. Li Q, Li GH, Liu L, Liu DL, Yu ZZ, Zuo M, et al. [Study on the relationship between nicotine dependence and depression among rural Dai older adults in Jinggu County, Yunnan Province]. Modern Preventive Medicine. (2024) 51(5):854-858. [in Chinese]. doi:10.20043/j.cnki.MPM.202310135.

# 38. Su HX, Li M, Li YW, Xie MH, Li SQ, Liang B. [A survey of anxiety and depression among older adults in a community of Bengbu city and analysis of influencing factors]. Chinese Journal of Clinical Healthcare. (2023) 26(6):787-791. [in Chinese].

# 39. Lin YM, Feng Y, Xue WC, Cao WX. [A survey on anxiety and depression among elderly people in rural areas along the Yellow River in Lüliang City, Shanxi Province]. Jiankang Jiaoyu yu Jiankang Cujin. (2023) 18(6):584-587. [in Chinese]. doi:10.16117/j.cnki.31-1974/r.202306584.

# 40. Yang J, Lv XZ, Shang L, Li HZ, Lu C, Zhang M, et al. [Detection rates and related factors of depression and anxiety among older adults in Shenzhen]. Chinese Mental Health Journal. (2023) 37(5):373-379. [in Chinese].

# 41. Ge HJ, Dong SH, Liu Y, Qi YT, Du J, Gao J, et al. [Identification of depression-related factors among older adults in China based on social determinants of health]. Modern Preventive Medicine. (2023) 50(22):4147-4152,4163. [in Chinese]. doi:10.20043/j.cnki.MPM.202305277.

# 42. Jiang Y, Lu Y, Cui J, Chu A. 24-hour movement behaviors time allocation and depression among Chinese community-dwelling older adults: a latent profile analysis. Geriatr Nurs. (2024) 58:382-387. doi:10.1016/j.gerinurse.2024.05.007

# 43. Zhang Y, Yu G, Bai W, Wu S, Geng X, Zhang W, et al. Association of depression and sleep quality with frailty: a cross-sectional study in China. Front Public Health. (2024) 12:1361745. doi:10.3389/fpubh.2024.1361745

# 44. Hu Q, Song Y, Wang S, Lin L, Ke Y, Zhang P. Association of subjective cognitive complaints with poor sleep quality: A cross-sectional study among Chinese elderly. Int J Geriatr Psychiatry. (2023) 38(6):e5956. doi:10.1002/gps.5956

# 45. Gan S, Yao X, Li Z. Correlation between social network and mental health in family-oriented older migrants: A study in two cities in China. Aging Health Res. (2024) 4(3):100201. doi:10.1016/j.ahr.2024.100201

# 46. Xiong H, Ma F, Tang D, Liu D. Correlations among nicotine dependence, health-related quality of life, and depression in current smokers: a cross-sectional study with a mediation model. Front Psychiatry. (2024) 15:1455918. doi:10.3389/fpsyt.2024.1455918

# 47. Peng X, Zhang S, You L, Hu W, Jin S, Wang J. Prevalence and correlates of depression and anxiety symptoms among older adults in Shenzhen, China: a cross-sectional population-based study. BMJ Open. (2024) 14(2):e077078. doi:10.1136/bmjopen-2023-077078

# 48. He ZF, Tan WY, Ma H, Shuai Y, Shan Z, Zhai J, et al. Prevalence and factors associated with depression and anxiety among older adults: A large-scale cross-sectional study in China. J Affect Disord. (2024) 346:135-143. doi:10.1016/j.jad.2023.11.022

# 49. Zeng H, Zhao W, Wang R, Li H, Wang L, Zeng X. Association between swallowing function and depressive symptoms among community-dwelling older adults: A cross-sectional study in Central China. J Affect Disord. (2025) 380:78-86. doi:10.1016/j.jad.2025.03.113

# 50. Wang X, Zhu B, Li JP, Li X, Zhang L, Wu Y, et al. The moderating effect of frailty on the network of depression, anxiety, and loneliness in community-dwelling older adults. J Affect Disord. (2025) 375:508-516. doi:10.1016/j.jad.2025.01.118

# 51. Chen YJ, Li Y, Wang H, Ding H. [The impact of anxiety and depression on sleep quality in rural older adults in China: the mediating role of social support]. Journal of Nanjing Medical University (Social Sciences). (2025) 25(1). [in Chinese].

# 52. Wu J, Xu H, Qin ZZ, Wang CC, Zhang Y, Hong X. [The effects of physical activity, depressive symptoms and their interaction on cognitive function among older adults in Nanjing]. Chinese Journal of Prevention and Control of Chronic Diseases. (2025) 33(1):29-32. [in Chinese]. doi:10.16386/j.cjpccd.issn.1004-6194.20240705.0485.

# 53. Zhang YX, Zhang SM, Jiang SQ, Chen SR, Li J, Zhou CC. [The relationship between adverse childhood experiences and depressive symptoms among rural older adults in Shandong Province]. Medicine and Society. (2025) 38(3):130-136. [in Chinese]. doi:10.13723/j.yxysh.2025.03.018.

# 54. Yang T, Liu Y, Li X, Gai Y, Xie Z, Wang J, et al. Association between household solid fuel use for cooking and depressive symptoms among middle-aged and elderly adults in rural China: Evidence from the China Family Panel Studies Database. J Environ Occup Med. (2025) 42(8):926-931. doi:10.5555/20250457080

# 55. Zhou L, Zhang A, Liu Q, Ji Q, Ye M, Xu Y, et al. Association Between Religious Service Attendance and Depression, Anxiety Among Older Amdo Tibetans. Int J Geriatr Psychiatry. (2025) 40(4):e70076. doi:10.1002/gps.70076

# 56. Du S, Lyu L, Cai Z, Wu Y, Zhou H. Association Between Sleep Quality and Depressive Symptoms in Community-Dwelling Older Adults: The Mediating Role of Loneliness. J Sichuan Univ Med Ed. (2025) 56(2):458. doi:10.12182/20250360605

# 57. Liu D, Yu Z, Liu L, Li G, Li X, Ruan C, et al. Socioeconomic disparities in the prevalence of depression and anxiety, and their associations with diabetes in rural southwest China. BMC Public Health. (2025) 25(1):668. doi:10.1186/s12889-025-21837-x

# 58. Tian W, Yan G, Zhang X, Shui D, Liu T, Yang J, et al. The prevalence, risk factors, awareness and treatment of depression and anxiety among adults aged 30 years and older in rural China: a cross-sectional study. J Affect Disord. (2025) 381:119516. doi:10.1016/j.jad.2025.119516

# 59. Wei SS, Liu KR, Wang RX, Tao J, He B, Wang YH. [Relationship of osteosarcopenia with depressive symptoms and physical activity in community-dwelling older adults]. Chinese Mental Health Journal. (2025) 39(7):611-616. [in Chinese].

# 60. Liu SX, Qi L, Chen C, Tan C, Qian H, Dai XL, et al. [Associations of lean body mass, fat mass and their indices with depressive symptoms among adults aged 65 years and above in 18 longevity areas of China]. National Medical Journal of China. (2025) 105(22):1840-1846. [in Chinese].

# 61. Liu CM, Liu AN, Liu MC. [Effects of intergenerational support and depression on social isolation in older adults]. Chinese Nursing Research. (2025) 39(8):1267-1271. [in Chinese].

# 62. Li D, Zuo PX, Duan YL, Yin FF, Cao YW. [Effects of social isolation and depression on cognitive function in community-dwelling older adults]. Journal of Nongken Medicine. (2025) 47(2):159-164. [in Chinese]. doi:10.27332/d.cnki.gshzu.2024.000329.

# 63. Zhao HR, Xiang XL, Wang ZJ, Xia Y, Ma M, Wang HD, et al. [Impact of social activities on quality of life among older adults: the chain mediating effect of depression and life satisfaction]. Modern Preventive Medicine. (2025) 52(10):1860-1866. [in Chinese]. doi:10.20043/j.cnki.MPM.202412498.

# 64. Feng W, Li ZX, Zhang W, Yuan J, He L, Li LJ, et al. [A study on the current status and influencing factors of cognitive decline and depressive state among the elderly in Karamay City]. Linchuang Yixue Jinzhan. (2025) 15(7):1350-1357. [in Chinese]. doi:10.12677/acm.2025.1572134

# 65. Lu J, Zhang F, Bao LM, Yang TL, Lu HL. [The effect of sedentary behavior on the incidence of depression among community-dwelling older adults in Ankang, Shaanxi]. Practical Preventive Medicine. (2025) 32(9):1126-1129. [in Chinese].

# 66. Chen RY, Li R, Li XW, Jiang ZD, Li YJ, Liu WL, et al. [Analysis on status and influencing factors of depressive symptoms among the elderly aged 60 years and above in Shandong Province]. Preventive Medicine Tribune. (2025) 31(6):433-437. [in Chinese]. doi:10.16406/j.pmt.issn.1672-9153.2025.6.07.

# 67. Dong P, Wu H, Chai Y, Huang P, Huang D, Lai L, et al. Impact of multimorbidity and cognitive impairment on depressive symptoms in community-dwelling older adults and their interaction effects. Sci Rep. (2025) 15(1):15033. doi:10.1038/s41598-025-99547-y

# 68. Dong P, Zhang X, Yin W, Shi Y, Xu M, Li H, et al. Relationship between short video addiction tendency and depression among rural older adults: cross-sectional study. J Med Internet Res. (2025) 27:e75938. doi:10.2196/75938

# 69. Wu S, Yang L, Mo H, Li L, Li Z, Ying Y. Prevalence and factors associated with anxiety and depression among the older people in ethnic minority areas in southern China: a cross-sectional study. Front Public Health. (2025) 13:1494629. doi:10.3389/fpubh.2025.1494629

# 70. Zhang XF, Yu LL, Zhang GH, Ma J, Qi C, Chen LX. [Depression status and its influencing factors among the population aged 65 years and above in Shandong Province]. Chinese Journal of Disease Control & Prevention. (2025) 29(12):1388-1393. [in Chinese]. doi:10.16462/j.cnki.zhjbkz.2025.12.004.

# 71. Lan Y, Yang JY, Shama SJ, Jiang Q, Qin JT, Wu Q, et al. [Study on the correlation between health-promoting lifestyles and depressive tendencies among older adults of Yi ethnic group in rural areas]. Soft Science of Health. (2025) 39(10):45-50. [in Chinese].

# 72. Wang YY, Bian YH, Gao B. [Actor-partner interdependence model analysis of the relationship between individual social capital and depressive symptoms among older couples across different socioeconomic statuses]. Journal of Sichuan University (Medical Sciences). (2025) 56(5):1344. [in Chinese].

# 73. Jing YR, Wang XN, Zhan GL, Zhang YS, Li XX, Wang Y. [Research on the association between socioeconomic status and mild cognitive impairment in community-dwelling older adults in a district of Shanghai]. Medicine and Society. (2025) 38(9). [in Chinese]. doi:10.13723/j.yxysh.2025.09.004.

# 74. Huang YY, Wang DH, Mao CX, Yi Y, Huang GY, Jiang SH, et al. [Study of the effect of self-perceived hearing status on depression in middle-aged and older people in the community]. Chinese Journal of Otorhinolaryngology Head and Neck Surgery. (2025) 60(9):1154-1162. [in Chinese].

# 75. Tian YH, Liu LW, Yang C, Ling C, Yang XX, Fan HJ, et al. [Associations and sex differences between depression and cognitive function among urban older adults]. Chinese General Practice. (2025) 28(1):47-52. [in Chinese].

# 76. Lin X, Huang LF, Chen Z, Liu YF, Gong FH. [A study on the correlation between depressive symptoms and lifestyles among rural older adults in Hunan Province]. Journal of Hunan Normal University (Medical Science). (2025) (3):198-204. [in Chinese].

# 77. Dong M, Wang SB, Tan WY, He HB, Hou CL. [Network analysis of depressive, anxiety, insomnia symptoms and cognitive function among community-dwelling older adults in Guangdong Province]. Chinese Journal of Psychiatry. (2026) 59(1):22-29. [in Chinese].

# 78. Zhang JY, Zhou ST, Li SF, Chen YM, Jia ZY, Wang YY, et al. [Effect of depressive symptoms on subjective well-being among younger older adults: the moderating role of subjective income level]. Journal of Humanities and Social Sciences. (2026) 2(4):142-148. [in Chinese]. doi:10.70693/rwsk.v2i4.402

# 79. Xu N, Ma X, Hu Q, Du J, He J, Wang W, et al. Association between multimorbidity and childhood socioeconomic status with depressive symptoms among middle-aged and older adults in rural western China. Sci Rep. (2026) 16:6600. doi:10.1038/s41598-026-37666-w

## 1.5 Supplementary Table 5. Subgroup random-effects meta-analysis of depressive symptom prevalence among older adults

| Variables | No. of Articles | No. of Participants | No. of Cases | I^2^(%) | Prevalence | 95% CI | χ^2^ | *P* |
| --- | --- | --- | --- | --- | --- | --- | --- | --- |
| Depressive Symptoms | 79 | 199116 | 35969 | 99.55 | 0.192 | 0.165 to 0.221 |  |  |
| Investigation year | | | | | | | 3.770 | 0.438 |
| 2020 | 20 | 45166 | 9942 | 99.56 | 0.178 | 0.129 to 0.241 |  |  |
| 2021 | 19 | 26138 | 5084 | 99.50 | 0.204 | 0.136 to 0.293 |  |  |
| 2022 | 20 | 70307 | 10291 | 99.62 | 0.166 | 0.121 to 0.224 |  |  |
| 2023 | 14 | 45308 | 8991 | 99.60 | 0.219 | 0.158 to 0.296 |  |  |
| 2024-2025 | 6 | 12197 | 3202 | 97.20 | 0.229 | 0.183 to 0.283 |  |  |
| Region | | | | | | | 9.970 | 0.019 |
| Eastern | 31 | 92775 | 12103 | 99.57 | 0.154 | 0.117 to 0.200 |  |  |
| Central | 10 | 25874 | 7191 | 99.29 | 0.277 | 0.212 to 0.355 |  |  |
| Western | 19 | 34156 | 7209 | 99.18 | 0.221 | 0.172 to 0.280 |  |  |
| Mixed | 19 | 46311 | 11007 | 99.58 | 0.190 | 0.140 to 0.253 |  |  |
| Measurement tool | | | | | | | 59.30 | <0.001 |
| PHQ | 29 | 118252 | 16433 | 99.67 | 0.157 | 0.120 to 0.202 |  |  |
| GDS | 28 | 37873 | 7501 | 98.86 | 0.168 | 0.135 to 0.208 |  |  |
| CES-D | 14 | 33013 | 11533 | 98.67 | 0.371 | 0.324 to 0.422 |  |  |
| Other | 8 | 9978 | 2043 | 99.10 | 0.165 | 0.100 to 0.260 |  |  |
| Gender | | | | | | | 2.750 | 0.097 |
| Male | 34 | 58180 | 9095 | 99.34 | 0.146 | 0.113 to 0.188 |  |  |
| Female | 34 | 65157 | 12859 | 99.57 | 0.199 | 0.153 to0.256 |  |  |
| Marriage | | | | | | | 4.160 | 0.041 |
| Married | 26 | 80944 | 12751 | 99.66 | 0.159 | 0.117 to 0.211 |  |  |
| Unmarried | 26 | 24332 | 5777 | 99.18 | 0.243 | 0.182 to 0.317 |  |  |
| Residence | | | | | | | 2.420 | 0.120 |
| Urban | 12 | 36917 | 3715 | 99.57 | 0.134 | 0.080 to 0.216 |  |  |
| Countryside | 21 | 42428 | 9223 | 99.45 | 0.209 | 0.065 to 0.270 |  |  |
| Living Statue | | | | | | | 4.520 | 0.034 |
| With other | 18 | 69013 | 8432 | 99.64 | 0.143 | 0.100 to 0.201 |  |  |
| Alone | 18 | 9902 | 1803 | 97.71 | 0.235 | 0.173 to 0.310 |  |  |
| Education Level | | | | | | | 7.220 | 0.007 |
| Junior secondary school or below | 17 | 41428 | 11292 | 99.32 | 0.268 | 0.216 to 0.327 |  |  |
| Senior secondary school and above | 13 | 8243 | 1408 | 97.88 | 0.149 | 0.100 to 0.217 |  |  |

The literature search was updated to 15 June 2026, whereas investigation years in the included studies ranged from 2020 to 2025.

χ² and P values refer to tests for differences between subgroups; Prevalence estimated using random-effects models;Abbreviations: CI = confidence interval; I^2^ = I-squared statistic.

## 1.6 Supplementary Table 6. Univariable random-effects meta-regression analyses of logit-transformed prevalence estimates

| Covariate | coefficient | 95% CI | P value |
| --- | --- | --- | --- |
| Investigation year | 0.058 | -0.106 to 0.222 | 0.488 |
| Region | | | |
| Eastern (Reference) |  |  |  |
| Central | 0.741 | 0.096 to 1.387 | 0.024 |
| Western | 0.441 | -0.076 to 0.958 | 0.341 |
| Mixed | 0.251 | -0.266 to 0.769 | 0.095 |
| Measurement tool | | | |
| PHQ (Reference) |  |  |  |
| CES-D | 1.161 | 0.631 to 1.691 | <0.001 |
| GDS | 0.082 | -0.351 to 0.515 | 0.710 |
| Other | 0.064 | -0.589 to 0.716 | 0.849 |

Coefficients are based on logit-transformed prevalence estimates.

## 1.7 Supplementary Table 7. Year-specific pooled odds ratios for sociodemographic disparities in depressive symptoms among older adults

| **Subgroup Variable** | **Investigation year** | **Category** | **No. of Articles** | **No. of Participants** | **Pooled OR** | **95% CI** | **I^2^(%)** | **P** |
| --- | --- | --- | --- | --- | --- | --- | --- | --- |
| **Gender** | 2020-2024 | Male | 34 | 58180 | 1.000 | - | - | - |
|  |  | Female |  | 65157 | 1.476 | 1.319 to 1.652 | 90.24 | <0.001 |
|  | 2020 | Male | 10 | 13872 | 1.000 | - | - | - |
|  |  | Female |  | 15061 | 1.437 | 1.117 to 1.848 | 92.98 | 0.005 |
|  | 2021 | Male | 7 | 6542 | 1.000 | - | - | - |
|  |  | Female |  | 7974 | 1.207 | 0.910 to 1.599 | 87.65 | <0.001 |
|  | 2022 | Male | 8 | 18217 | 1.000 | - | - | - |
|  |  | Female |  | 20635 | 1.574 | 1.143 to 2.168 | 89.18 | <0.001 |
|  | 2023 | Male | 6 | 14797 | 1.000 | - | - | - |
|  |  | Female |  | 16273 | 1.760 | 1.498 to 2.067 | 83.64 | <0.001 |
|  | 2024 | Male | 3 | 4752 | 1.000 | - | - | - |
|  |  | Female |  | 5214 | 1.422 | 1.013 to 1.997 | 89.10 | <0.001 |
|  | Between | |  |  |  |  |  | 0.198 |
| **Marriage** | 2020-2024 | Married | 26 | 47179 | 1.000 | - | - | - |
|  |  | Unmarried |  | 15589 | 1.652 | 1.467 to 1.860 | 86.59 | <0.001 |
|  | 2020 | Married | 6 | 11793 | 1.000 | - | - | - |
|  |  | Unmarried |  | 3210 | 1.590 | 1.363 to 1.856 | 52.43 | 0.062 |
|  | 2021 | Married | 5 | 8555 | 1.000 | - | - | - |
|  |  | Unmarried |  | 3375 | 1.706 | 1.033 to 2.817 | 92.67 | <0.001 |
|  | 2022 | Married | 8 | 30926 | 1.000 | - | - | - |
|  |  | Unmarried |  | 7926 | 1.619 | 1.271 to 2.063 | 78.28 | <0.001 |
|  | 2023 | Married | 4 | 21840 | 1.000 | - | - | - |
|  |  | Unmarried |  | 7685 | 1.669 | 1.203 to 2.317 | 96.05 | <0.001 |
|  | 2024 | Married | 3 | 7830 | 1.000 | - | - | - |
|  |  | Unmarried |  | 2136 | 1.732 | 1.558 to 1.860 | 0.00 | 0.373 |
|  | Between | |  |  |  |  |  | 0.925 |
| **Residence** | 2020-2024 | Urban | 12 | 36917 | 1.000 | - | - | - |
|  |  | Rural |  | 22437 | 1.419 | 1.185 to 1.699 | 89.78 | <0.001 |
|  | 2020 | Urban | 2 | 2779 | 1.000 | - | - | - |
|  |  | Rural |  | 2860 | 1.680 | 1.486 to 1.899 | 0.00 | 0.907 |
|  | 2021 | Urban | 4 | 3826 | 1.000 | - | - | - |
|  |  | Rural |  | 4916 | 1.300 | 0.786 to 2.149 | 91.61 | <0.001 |
|  | 2022 | Urban | 3 | 25862 | 1.000 | - | - | - |
|  |  | Rural |  | 9446 | 1.305 | 1.010 to 1.685 | 81.48 | 0.005 |
|  | 2023-2024 | Urban | 3 | 4450 | 1.000 | - | - | - |
|  |  | Rural |  | 5215 | 1.419 | 1.185 to 1.699 | 95.50 | <0.001 |
|  | Between | |  |  |  |  |  | 0.296 |
| **Living Status** | 2020-2024 | With others | 18 | 69013 | 1.000 | - | - | - |
|  |  | Alone |  | 9902 | 1.671 | 1.437 to 1.945 | 79.09 | <0.001 |
|  | 2020 | With others | 4 | 2702 | 1.000 | - | - | - |
|  |  | Alone |  | 317 | 2.858 | 1.222 to 6.686 | 83.60 | <0.001 |
|  | 2021 | With others | 3 | 6826 | 1.000 | - | - | - |
|  |  | Alone |  | 1303 | 1.828 | 0.794 to 4.206 | 95.11 | <0.001 |
|  | 2022 | With others | 6 | 34273 | 1.000 | - | - | - |
|  |  | Alone |  | 3387 | 1.763 | 1.417 to 2.192 | 56.26 | 0.043 |
|  | 2023 | With others | 2 | 17868 | 1.000 | - | - | - |
|  |  | Alone |  | 3806 | 1.403 | 1.225 to 1.607 | 51.80 | 0.150 |
|  | 2024 | With others | 3 | 7344 | 1.000 | - | - | - |
|  |  | Alone |  | 1089 | 1.387 | 1.209 to 1.591 | 0.00 | 0.787 |
|  | Between | |  |  |  |  |  | 0.170 |
| **Education Level** | 2020-2024 | Senior secondary school and above | 11 | 7280 | 1.000 | - | - | - |
|  |  | Junior secondary school or below |  | 31713 | 1.693 | 1.388 to 2.065 | 80.84 | <0.001 |
|  | 2020 | Senior secondary school and above | 6 | 3140 | 1.000 | - | - | - |
|  |  | Junior secondary school or below |  | 9022 | 1.789 | 1.292 to 2.475 | 84.59 | <0.001 |
|  | 2021 | Senior secondary school and above | 2 | 515 | 1.000 | - | - | - |
|  |  | Junior secondary school or below |  | 4614 | 1.297 | 0.924 to 1.821 | 22.48 | 0.256 |
|  | 2023-2024 | Senior secondary school and above | 3 | 3625 | 1.000 | - | - | - |
|  |  | Junior secondary school or below |  | 18077 | 1.812 | 1.207 to 2.721 | 84.60 | 0.002 |
|  | Between | |  |  |  |  |  | 0.319 |

The literature search was updated to 15 June 2026, whereas investigation years in the included studies ranged from 2020 to 2024.

Abbreviations: CI = confidence interval; I^2^ = I-squared statistic.

Year-specific pooled ORs were presented only when sufficient subgroup-specific data were available; therefore, later-year strata differed across subgroup variables.

Estimates based on 2–3 studies should be interpreted cautiously.

For each year stratum, P values refer to the pooled OR test within strata; the “Between” row reports the p value for heterogeneity across year strata.

When Cochran’s Q was smaller than its degrees of freedom, I² was truncated at 0.

## 1.8 Supplementary Table 8. Year-level inverse-variance weighted trend regression of year-specific pooled odds ratios (ORs), 2020–2024

| **Subgroup Variable** | **exp(β) (per 1-year increase)** | **95% CI** | **P value** | **No. of years (n)** |
| --- | --- | --- | --- | --- |
| **Gender**  **(Female vs Male)** | 1.062 | 0.920 to 1.225 | 0.275 | 5 |
| **Marital status**  **(Unmarried vs Married)** | 1.021 | 1.016 to 1.026 | 0.001 | 5 |
| **Residence**  **(Rural vs Urban)** | 0.937 | 0.857 to 1.025 | 0.089 | 4 |
| **Living arrangement**  **(Alone vs With others)** | 0.892 | 0.788 to 1.009 | 0.060 | 5 |
| **Education Level**  **(Junior secondary school or below vs Senior secondary school and above)** | 1.014 | 0.321 to 3.196 | 0.906 | 3 |

Abbreviations. For each comparison, ORs were pooled within each investigation year using random-effects models. The natural logarithm of the year-specific pooled OR [ln(OR)] was regressed on investigation year using inverse-variance weights (w = 1/SE²), where SE was reconstructed from the 95% CI of the pooled ln(OR): SE = [ln(UCI) − ln(LCI)]/(2×1.96). Robust standard errors were used. exp(β) represents the multiplicative change in pooled OR per one-year increase in investigation year.

## 1.9 Supplementary Table 9. Leave-one-out sensitivity analysis of the influence of a single study on the pooled prevalence of depression among older adults

| Number | Study Omitted | Estimate | 95%CI (%) |
| --- | --- | --- | --- |
| 1 | Chen B (2023) | -1.428 | [-1.608,-1.247] |
| 2 | Qin Z (2022) | -1.437 | [-1.619,-1.256] |
| 3 | Yu J (2022) | -1.453 | [-1.634,-1.272] |
| 4 | Qin Y (2022) | -1.429 | [-1.610,-1.249] |
| 5 | Liu J (2022) | -1.454 | [-1.635,-1.273] |
| 6 | Cui (2022) | -1.439 | [-1.619,-1.258] |
| 7 | Xu K (2022) | -1.446 | [-1.627,-1.265] |
| 8 | Liu C (2022) | -1.453 | [-1.634,-1.272] |
| 9 | Lu C (2022) | -1.452 | [-1.633,-1.270] |
| 10 | Lu L (2023) | -1.452 | [-1.633,-1.271] |
| 11 | Hao R (2023) | -1.457 | [-1.637,-1.277] |
| 12 | Li Y (2022) | -1.457 | [-1.637,-1.278] |
| 13 | Li R (2022) | -1.434 | [-1.615,-1.253] |
| 14 | Li S (2022) | -1.431 | [-1.612,-1.250] |
| 15 | Ding K (2022) | -1.438 | [-1.619,-1.257] |
| 16 | Hou P (2022) | -1.430 | [-1.611,-1.250] |
| 17 | Mao S (2022) | -1.472 | [-1.651,-1.294] |
| 18 | Liu W (2022) | -1.421 | [-1.601,-1.241] |
| 19 | Wang S (2021) | -1.436 | [-1.618,-1.254] |
| 20 | Liu A (2021) | -1.435 | [-1.616,-1.254] |
| 21 | Zhou R (2021) | -1.435 | [-1.616,-1.254] |
| 22 | Liang W (2021) | -1.448 | [-1.629,-1.267] |
| 23 | Wang G (2020) | -1.427 | [-1.607,-1.246] |
| 24 | Wang S (2020) | -1.431 | [-1.612,-1.250] |
| 25 | He L (2022) | -1.448 | [-1.630,-1.266] |
| 26 | Li H (2022) | -1.425 | [-1.606,-1.245] |
| 27 | Yu H (2024) | -1.404 | [-1.582,-1.225] |
| 28 | Zhao Z (2024) | -1.437 | [-1.619,-1.255] |
| 29 | Liu M (2024) | -1.435 | [-1.616,-1.254] |
| 30 | Zong L (2022) | -1.423 | [-1.603,-1.244] |
| 31 | Zhang Y (2024) | -1.454 | [-1.632,-1.275] |
| 32 | Liu Y (2023) | -1.427 | [-1.608,-1.246] |
| 33 | Yang L (2024) | -1.458 | [-1.637,-1.279] |
| 34 | Liu S (2024) | -1.443 | [-1.624,-1.262] |
| 35 | Huang W (2024) | -1.450 | [-1.633,-1.267] |
| 36 | Zhao Q (2023) | -1.450 | [-1.631,-1.269] |
| 37 | Li Q (2024) | -1.459 | [-1.638,-1.281] |
| 38 | Su H (2023) | -1.439 | [-1.620,-1.258] |
| 39 | Lin Y (2023) | -1.431 | [-1.612,-1.251] |
| 40 | Yang J (2023) | -1.448 | [-1.629,-1.265] |
| 41 | Ge H (2023) | -1.447 | [-1.631,-1.264] |
| 42 | Jiang Y (2024) | -1.441 | [-1.622,-1.259] |
| 43 | Zhang Y (2024) | -1.435 | [-1.616,-1.254] |
| 44 | Hu Q (2023) | -1.435 | [-1.616,-1.254] |
| 45 | Gan S (2024) | -1.437 | [-1.618,-1.256] |
| 46 | Xiong H (2024) | -1.441 | [-1.622,-1.260] |
| 47 | Peng X (2024) | -1.431 | [-1.611,-1.250] |
| 48 | He Z (2024) | -1.411 | [-1.586,-1.236] |
| 49 | Zeng H (2025) | -1.438 | [-1.621,-1.254] |
| 50 | Wang X (2025) | -1.453 | [-1.634,-1.272] |
| 51 | Chen Y (2025) | -1.459 | [-1.638,-1.279] |
| 52 | Wu J (2025) | -1.434 | [-1.615,-1.252] |
| 53 | Zhang Y (2025) | -1.441 | [-1.624,-1.259] |
| 54 | Yang T (2025) | -1.451 | [-1.632,-1.269] |
| 55 | Zhou L (2025) | -1.456 | [-1.637,-1.276] |
| 56 | Du S (2025) | -1.448 | [-1.629,-1.266] |
| 57 | Liu D (2025) | -1.426 | [-1.606,-1.246] |
| 58 | TianW (2025) | -1.425 | [-1.605,-1.245] |
| 59 | Wei S (2025) | -1.441 | [-1.622,-1.259] |
| 60 | Liu S (2025) | -1.422 | [-1.601,-1.243] |
| 61 | Liu C (2025) | -1.446 | [-1.627,-1.264] |
| 62 | Li D (2025) | -1.446 | [-1.628,-1.264] |
| 63 | Zhao H (2025) | -1.443 | [-1.624,-1.262] |
| 64 | Feng W (2025) | -1.436 | [-1.620,-1.252] |
| 65 | Lu J (2025) | -1.440 | [-1.621,-1.259] |
| 66 | Chen R (2025) | -1.443 | [-1.625,-1.261] |
| 67 | Dong P (2025) | -1.445 | [-1.632,-1.257] |
| 68 | Dong P (2025) | -1.446 | [-1.627,-1.265] |
| 69 | Wu S (2025) | -1.437 | [-1.618,-1.256] |
| 70 | Zhang X (2025) | -1.421 | [-1.587,-1.255] |
| 71 | Lan Y (2025) | -1.440 | [-1.621,-1.259] |
| 72 | Wang Y (2025) | -1.452 | [-1.633,-1.271] |
| 73 | Jing Y (2025) | -1.427 | [-1.607,-1.247] |
| 74 | Huang Y (2025) | -1.430 | [-1.611,-1.250] |
| 75 | Tian Y (2025) | -1.436 | [-1.617,-1.255] |
| 76 | Lin X (2025) | -1.448 | [-1.632,-1.264] |
| 77 | Dong M (2026) | -1.430 | [-1.611,-1.250] |
| 78 | Zhang J (2026) | -1.445 | [-1.628,-1.261] |
| 79 | Xu N (2026) | -1.443 | [-1.625,-1.260] |

Abbreviations: CI = confidence interval.

## 1.10 Supplementary Table 10. Sensitivity analysis of year-stratified prevalence estimates using the Freeman–Tukey double arcsine transformation

| Investigation year | No. of studies | Primary analysis prevalence | 95%CI | Freeman-Tukey prevalence | 95%CI |
| --- | --- | --- | --- | --- | --- |
| 2020 | 20 | 0.178 | 0.129 to 0.241 | 0.200 | 0.140 to 0.280 |
| 2021 | 19 | 0.204 | 0.136 to 0.293 | 0.220 | 0.150 to 0.310 |
| 2022 | 20 | 0.166 | 0.121 to 0.224 | 0.180 | 0.120 to 0.240 |
| 2023 | 14 | 0.219 | 0.158 to 0.296 | 0.240 | 0.170 to 0.300 |
| 2024–2025 | 6 | 0.229 | 0.183 to 0.283 | 0.230 | 0.180 to 0.290 |

The Freeman–Tukey double arcsine transformation was used as a sensitivity analysis for prevalence pooling. Estimates were slightly higher but broadly consistent with the primary analysis and did not alter the observed year-level pattern.

2 Supplementary Figures

## 2.1 Supplementary Figure 1. Year-stratified pooled odds ratios for depressive symptoms in older adults with junior secondary school or below compared with those with senior secondary school and above, 2020-2024

The literature search was updated until 15 June 2026. However, the analyses in the present study were based on investigations conducted between 2020 and 2024. SSA and JSB were used as abbreviations for senior secondary school and above and junior secondary school or below, respectively. Estimates for 2023 and 2024 were combined because year-specific data were sparse; points are shown without a connecting line to avoid implying continuity across years with missing estimates.

## 2.2 Supplementary Figure 2. Year-stratified pooled odds ratios for depressive symptoms in older adults living alone compared with those living with others, 2020-2024

The literature search was updated until 15 June 2026. However, the analyses in the present study were based on investigations conducted between 2020 and 2024.

## 2.3 Supplementary Figure 3. Year-stratified pooled odds ratios for depressive symptoms in unmarried older adults compared with married older adults, 2020-2024

The literature search was updated until 15 June 2026. However, the analyses in the present study were based on investigations conducted between 2020 and 2024.

## 2.4 Supplementary Figure 4. Year-stratified pooled odds ratios for depressive symptoms in female older adults compared with male older adults, 2020-2024

The literature search was updated until 15 June 2026. However, the analyses in the present study were based on investigations conducted between 2020 and 2024.

## 2.5 Supplementary Figure 5. Year-stratified pooled odds ratios for depressive symptoms in rural older adults compared with urban older adults, 2020-2024

The literature search was updated until 15 June 2026. However, the analyses in the present study were based on investigations conducted between 2020 and 2024. Estimates for 2023 and 2024 were combined because year-specific data were sparse.
